# Mitochondria-targeted accumulation of oxygen-irrelevant free radicals for enhanced synergistic low-temperature photothermal and thermodynamic therapy

**DOI:** 10.1186/s12951-021-01142-6

**Published:** 2021-11-25

**Authors:** Hongzhi Hu, Xiangtian Deng, Qingcheng Song, Wenbo Yang, Yiran Zhang, Weijian Liu, Shangyu Wang, Zihui Liang, Xin Xing, Jian Zhu, Junzhe Zhang, Zengwu Shao, Baichuan Wang, Yingze Zhang

**Affiliations:** 1 Department of Orthopaedics, Union Hospital, Tongji Medical College, Huazhong University of Science and Technology, Wuhan, 430022 China; 2 School of Medicine, Nankai University, Tianjin, 300071 China; 3 Department of Orthopaedic Surgery, The Third Hospital of Hebei Medical University, Shijiazhuang, 050051 China; 4 NHC Key Laboratory of Intelligent Orthopeadic Equipment, Third Hospital of Hebei Medical University, Shijiazhuang, Hebei China; 5 Collaborative Innovation Center for Advanced Organic Chemical Materials Co-Constructed by the Province and Ministry, Hubei University, Wuhan, 430062 China

**Keywords:** Low temperature photothermal therapy, Thermodynamic therapy, Mitochondria-targeting, MnO_2_ nanoparticle, Oxygen-irrelevant free radicals, Azo initiator

## Abstract

**Background:**

Although lower temperature (< 45 °C) photothermal therapy (LPTT) have attracted enormous attention in cancer therapy, the therapeutic effect is still unsatisfying when applying LPTT alone. Therefore, combining with other therapies is urgently needed to improve the therapeutic effect of LPTT. Recently reported oxygen-irrelevant free radicals based thermodynamic therapy (TDT) exhibit promising potential for hypoxic tumor treatment. However, overexpression of glutathione (GSH) in cancer cells would potently scavenge the free radicals before their arrival to the specific site and dramatically diminish the therapeutic efficacy.

**Methods and results:**

In this work, a core–shell nanoplatform with an appropriate size composed of arginine–glycine–aspartate (RGD) functioned polydopamine (PDA) as a shell and a triphenylphosphonium (TPP) modified hollow mesoporous manganese dioxide (H-mMnO_2_) as a core was designed and fabricated for the first time. This nanostructure endows a size-controllable hollow cavity mMnO_2_ and thickness-tunable PDA layers, which effectively prevented the pre-matured release of encapsulated azo initiator 2,2′-azobis[2-(2-imidazolin-2-yl) propane] dihydrochloride (AIBI) and revealed pH/NIR dual-responsive release performance. With the mitochondria-targeting ability of TPP, the smart nanocomposites (AIBI@H-mMnO_2_-TPP@PDA-RGD, AHTPR) could efficiently induce mitochondrial associated apoptosis in cancer cells at relatively low temperatures (< 45 °C) via selectively releasing oxygen-irrelevant free radicals in mitochondria and facilitating the depletion of intracellular GSH, exhibiting the advantages of mitochondria-targeted LPTT/TDT. More importantly, remarkable inhibition of tumor growth was observed in a subcutaneous xenograft model of osteosarcoma (OS) with negligible side effects.

**Conclusions:**

The synergistic therapy efficacy was confirmed by effectively inducing cancer cell death in vitro and completely eradicating the tumors in vivo. Additionally, the excellent biosafety and biocompatibility of the nanoplatforms were confirmed both in vitro and in vivo. Taken together, the current study provides a novel paradigm toward oxygen-independent free-radical-based cancer therapy, especially for the treatment of hypoxic solid tumors.

**Graphical Abstract:**

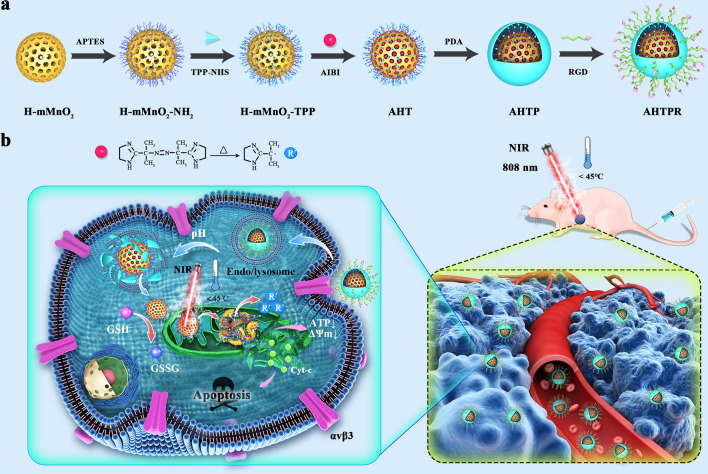

**Supplementary Information:**

The online version contains supplementary material available at 10.1186/s12951-021-01142-6.

## Introduction

Photothermal therapy (PTT) has recently emerged as a promising cancer therapeutic strategy due to its merits of noninvasiveness, specific spatiotemporal selectivity, and negligible drug resistance [1, 2]. Generally, PTT heavily depends on a high power intensity near-infrared laser (NIR) irradiation to ensure therapeutic efficacy, which inevitably damages the nearby normal tissues [3]. Recently, developing a PTT strategy with relatively low temperatures (< 45 °C) to realize effective tumor destruction can be very important for clinical translation in the future [4, 5]. However, the therapeutic effect is still unsatisfying when applying low-temperature PTT (LPTT) alone, which is ascribed to inducible heat resistance of cancer cells, poor penetration of exogenous light stimulation, and low accumulation of photothermal agents in tumor sites [6]. Therefore, it is highly desirable to combine with other therapies to improve the therapeutic effect of LPTT.

Reactive oxygen species (ROS)-based cancer dynamic therapy, such as chemodynamic therapy (CDT) [7], photodynamic therapy (PDT) [8], sonodynamic therapy (SDT) [9], has attracted tremendous attention in recent years. Unfortunately, the performance of these oxygen-dependent anticancer modalities is seriously compromised by the hypoxic tumor microenvironment at solid tumors [4]. In accordance with the problem, enormous efforts have been made including intra-tumoral oxygen delivery [10] or oxygen production in situ [11]. Nevertheless, these strategies still could not move away from the dependence on oxygen fundamentally. In this regard, an ingenious strategy by using oxygen-irrelevant free radicals is expected to achieve effective antitumor thermodynamic therapy (TDT) under mild thermal stimulation [4, 12, 13]. Thermal-labile azo initiators, such as azo initiator 2,2′-azobis[2-(2-imidazolin-2-yl) propane] dihydrochloride (AIBI), can be decomposed rapidly into highly reactive alkyl radicals under mild heat stimulation without oxygen participation [13, 14]. However, the efficacy induced by the free radicals is spatially and temporally restricted due to their short lifetime and confined diffusion distance [15]. Endeavoring free radicals with subcellular organelle targeting ability might dramatically enhance their therapeutic efficacy. Thus, selectively generation of free radicals in mitochondria may significantly enhance the efficacy of the TDT because mitochondria are susceptible to free-radical mediated damages [15]. Unfortunately, intracellular overexpression of glutathione (GSH) in cancer cells, which might potently inactive the alkyl radicals, dramatically diminished the therapeutic efficacy [16]. Therefore, we intend to develop a smart therapy strategy, which can not only selectively accumulate free radicals in mitochondria but also simultaneously consume the intracellular GSH, to achieve synergistically enhanced LPTT/TDT.

Manganese dioxide (MnO_2_) nanostructures have recently aroused great interest as a unique type of TME-responsive nanomaterial [17]. MnO_2_ NPs can facilitate GSH oxidation to yield glutathione disulfide (GSSG), thus effectively depleting the intracellular GSH [18, 19]. In addition, MnO_2_ NPs exhibited excellent photothermal conversion efficiency, with great potential to completely ablate tumors under NIR irradiation. However, most previously reported MnO_2_ nanostructures are in the form of nanosheets and nanoparticles, which may not be ideal to realize drug delivery or accurately controlling drug release [20, 21]. Recently, hollow mesoporous MnO_2_ (H-mMnO_2_) nanostructures have been extensively studied as an excellent drug loading/delivery system due to their loading efficiency and prominent biodegradability [18, 22, 23]. In order to achieve “zero release”, one promising strategy is to block the pores of H-mMnO_2_ using gatekeepers. Polydopamine (PDA), a mussel-inspired material, has strong adhesion so that it could be coated on the surface of various materials under slightly alkaline conditions [24]. Of note, PDA shell could be depolymerized to release encapsulated drugs in an acid environment, avoiding undesirable premature drug leakage during circulation in the blood [23]. In addition, the rich chemical groups on the PDA surface enable it to achieve further functionalization. With these findings in mind, we hypothesized PDA coated H-mMnO_2_ NPs with versatile designs have the potential to achieve precisely targeted delivery of azo initiators while efficiently depleting intracellular GSH.

**Scheme 1:**
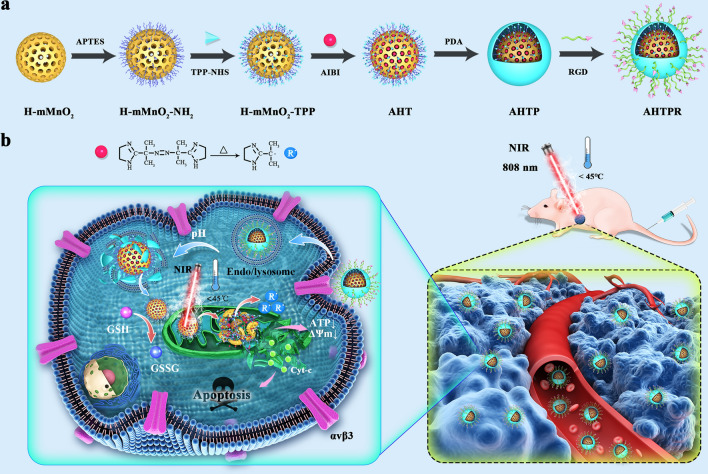
**a** Schematic illustration of the synthesis procedures of AHTPR NPs. **b** The application of the NPs in the synergistic mitochondria-targeted LPTT/TDT

Herein, we, for the first time, rationally designed an intelligent core–shell nanoplatform based on H-mMnO_2_ for tumor-targeted drug delivery, pH-triggered controllable release and mitochondrial targeting accumulation of free radicals, so as to achieve synergistically enhanced LPTT/TDT. As illustrated in Scheme 1a, H-mMnO_2_ NPs were firstly prepared as previously described with mild modification. Mitochondria-targeting ligands triphenylphosphine (TPP) was then modified on the surface of H-mMnO_2_ to construct the H-mMnO_2_-TPP. Subsequently, the azo initiator AIBI was encapsulated into H-mMnO_2_-TPP and coated with PDA (AIBI@H-mMnO_2_-TPP@PDA, AHTP), which could pervert the premature leakage of AIBI due to the excellent stability of PDA shell at pH 7.4. In this study, we chose osteosarcoma (OS), the most common malignant solid tumor that affects bones [25], as a treatment model. Arginine–glycine–aspartate (RGD), a cell-affinitive peptide can interact with αvβ3 and αvβ5 integrin receptors, which are widely expressed in various cancer cells [26–28], including OS cell lines such as MNNG/HOS and MG-63 cells [29, 30]. Therefore, RGD with specific tumor-targeting properties was finally conjugated onto AHTP to obtain AIBI@H-mMnO_2_-TPP@PDA-RGD (AHTPR) NPs. As shown in Scheme 1b, surface modified RGD could promote AHTPR NPs specific targeting to OS cells. After the PDA shell was destroyed in the acidic tumor environment, the NPs could accumulate in mitochondria through the TPP targeting ability. With the irradiation of NIR light, the NPs could generate low temperature locally, which might subsequently stimulate the thermal decomposition of AIBI to induce free radicals burst. More importantly, the intracellular GSH could be oxidized into GSSG by MnO_2_, which significantly decreased the consumption of the alkyl radicals. Consequently, a large number of free radicals rapidly accumulated in mitochondria, decreased mitochondrial membrane potential, and ultimately provoked the mitochondria-mediated cell apoptosis. Taken together, this work provided an innovative anticancer therapeutic strategy to achieve synergistically enhanced mitochondrial targeting LPTT/TDT.

## Methods

### Materials

Tetraethyl orthosilicate (TEOS), dopamine hydrochloride, anhydrous ethanol, ammonium hydroxide (NH_3_·H_2_O), N-hydroxysuccinimide (NHS), N-(3-dimethylaminopropyl)-N-ethylcarbodiimide hydrochloride (EDC), and triphenylphosphine (TPP) were obtained from Sigma-Aldrich (St. Louis, MO, USA). Potassium permanganate (KMnO_4_, 99%), and sodium carbonate (Na_2_CO_3_), were obtained from Sinopharm Chemical Reagent Co., Ltd. (Shanghai, China). 2,2’-azobis[2-(2-imidazolin-2-yl) propane] dihydrochloride (AIBI) was purchased from Macklin Biochemical Co., Ltd. (Shanghai, China). Primary antibodies against Bcl-2 (Cat No. GTX100064), Bax (Cat No. GTX109683), Cytochrome c (Cyt-c, Cat No. GTX108585), cleaved-Caspase-9 (Cat No. GTX22324), cleaved-Caspase-3 (Cat No. GTX22302) and GAPDH (Cat No. GTX100118) were purchased from GeneTex (Irvine, CA, USA). All other chemicals and reagents were of the highest quality commercially available and used as received.

### Synthesis of hollow mesoporous MnO_2_ (H-mMnO_2_)

H-mMnO2 NPs were synthesized as previously described with mild modification [31]. In brief, solid SiO_2_ NPs (sSiO_2_) were first synthesized according to the following route: 2 mL of TEOS was added dropwise to a mixed solution containing 12 mL of ethanol, 3 mL of deionized water, and 1 mL NH_3_·H_2_O, and stirred at 50 °C for 2 h. Subsequently, the sSiO_2_ NPs were collected by centrifugation at 10,000 rpm for 10 min, and then washed by ethanol and DI water three times, respectively.

For the synthesis of sSiO_2_@MnO_2_ NPs, an aqueous solution of KMnO_4_ (500 mg) was dropwise added into the above sSiO_2_ aqueous suspension under ultrasonication. After stirring for 12 h, the precipitate obtained by centrifugation at 10,000 rpm for 10 min and washed with DI water for three times. To etch the inner SiO_2_ template, the as-prepared sSiO_2_@MnO_2_ NPs was dispersed in a Na_2_CO_3_ aqueous solution and heated at 60 °C for 12 h. Finally, the obtained hollow mesoporous MnO_2_ (H-mMnO_2_) products were collected by centrifugation and washed with DI water for three times.

### Synthesis of H-mMnO_2_-TPP

For the synthesis of the TPP modified H-mMnO_2_ (H-mMnO_2_-TPP), the surface of H-mMnO_2_ was first functionalized with amine groups. 75 mg H-mMnO_2_ were dispersed in methylbenzene (60 mL) with the help of ultrasonication. Then, 250 µL of APTES was added dropwise to the solution and heated to 80 °C in N_2_ environment and kept for 12 h. After that, the obtained amino-functionalized H-mMnO_2_ (H-mMnO_2_-NH_2_) were collected by centrifugation and washed several times with ethanol to remove the remaining solvent and rinsed by deionized water for three times.

To prepare H-mMnO_2_-TPP, TPP-NHS was firstly synthesized following the previously reported method [32]. Then, 10 mg TPP-NHS was dissolved under stirring in 10 mL of DMSO and then mixed with 10 mg H-mMnO_2_ under ultrasonication for 30 min. Then, the mixture was stirred at room temperature for 24 h. After centrifugation at 10,000 rpm for 10 min, the resulting H-mMnO_2_-TPP NPs were collected and washed with DI water for three times.

### Preparation of AIBI@H-mMnO_2_-TPP@PDA (AHTP)

For AIBI loading, 5 mL as-synthesized H-mMnO_2_-TPP solution (2 mg mL^−1^) was dispersed in 20 mL AIBI aqueous solution (2 mg mL^−1^) and stirred overnight at room temperature in darkness. The solution was then centrifuged at 12,000 rpm for 10 min and washed three times DI with water to remove the remaining AIBI. The resulting products (AIBI@H-mMnO_2_-TPP, AHT) were dispersed in 5 mL water for further use.

The obtained AHT NPs were dispersed in 10 mL Tris–HCl buffer (pH = 8.5, 10 mM) containing dopamine hydrochloride (10 mg) and stirred at room temperature for 6 h. Afterwards, the prepared PDA-coated NPs (AIBI@H-mMnO_2_-TPP@PDA, AHTP) were collected by centrifugation and washed with DI water for three times.

### Synthesis of AIBI@H-mMnO_2_-TPP@PDA-RGD (AHTPR)

Briefly, 5 mg RGD was dispersed in DMSO (2 mL) and then mixed with 2 mL AHTP solution (2 mg mL^−1^), which was stirred overnight at room temperature in the dark. Thereafter, the resultants were rinsed in DI water three times by centrifugation (10,000 rpm). Finally, the obtained purified AIBI@H-mMnO_2_-TPP@PDA-RGD (AHTPR) NPs were stored in the dark for further characterization and application.

### Characterizations

Scanning electron microscope (SEM) images were taken on a transmission electron microscope (ZEISS Gemini 300). Transmission electron microscopy (TEM) images were recorded on a JEOL JEM F200 electron microscope operating at 200 kV. Dynamic light scattering (DLS) and zeta potential were evaluated by using a Zetasizer Nano ZS90 equipment (Malvern Instruments, UK). Fourier transform infrared (FTIR) spectra were scanned on a Thermo Nicolet iS50 FTIR spectrometer in the range of 400–4000 cm^−1^. UV–vis absorption spectra was evaluated with a spectrophotometer (UV-3600 Shimadzu, Japan). The X-ray photoelectronic spectroscopy (XPS) spectra were recorded with a spectrophotometer (Thermo Scientific K-Alpha). The Brunauer–Emmett–Teller (BET) approach (ASAP 2460, Micromeritics, USA) was used to investigate the surface area and pore size distributions of the samples. Electron paramagnetic resonance (EPR) spectroscopy spectrums were obtained from a Bruker EMXnano Spectrometer.

### In vitro photothermal performance

To determine the photothermal performance of AHTPR NPs in vitro, the temperature variation was monitored in real-time and recorded at the designed time intervals using an infrared thermal imaging camera (Testo 865, Testo, Schwarzwald, Germany). To investigate the concentration and time-dependency of the photothermal effect, various concentrations of AHTPR NPs in PBS (0, 30, 60, and 120 μg mL^−1^) were irradiated with an 808 nm laser (1.0 W cm^−2^) for 10 min. To further examine the laser power density-dependency of the photothermal effect, 60 ug mL^−1^ samples were irradiated at different power densities (0.5, 1.0, 1.5, and 2.0 W cm^−2^) for 10 min. In addition, the photothermal stability of AHTPR NPs were study by five cycles of ON/OFF NIR-laser irradiation. In brief, the samples were irradiated for 10 min, and then cooled down to room temperature prior to the next cycle.

### Hemolysis assay

Briefly, 1 mL anticoagulated whole blood samples were obtained from the orbital venous plexus of health Kunming mice and then centrifuged at 8000*g* at 4 °C for 5 min to isolate red blood cells (RBCs). Afterward, RBCs were washed five times and diluted with 2 mL PBS. RBC diluted suspensions (200 µL) were added into 800 µL of deionized water (positive control), PBS (negative control), or various concentration of AHTPR NPs (50–1600 µg mL^−1^). After 2 h incubation at 37 °C in a shaker table, the mixtures were centrifuged at 8000*g* at 4 °C for 5 min. Subsequently, the absorbance of all gained supernatant was measured at 541 nm. In the end, the RBCs’ hemolysis percentage was calculated by using the following equation: Hemolysis percentages (%) = (*I*_sample_ – *I*_negative control_) / (*I*_positive control_ – *I*_negative control_) × 100%, where *I*_sample_, *I*_negative control_, *I*_positive control_, and *I*_negative control_ represent the absorbance of the sample, negative control, and positive control, respectively.

### Drug release in vitro

The dialysis method was utilized to analyze the triggered release behavior of AIBI from AHTPR NPs in different environments. Briefly, 1 mL AHTPR (2 mg/mL) NPs was dialyzed against PBS (30 mL) at different pH values (5.0 and 7.4) with or without exposure to NIR laser irradiation (1.0 W cm^−2^) for 10 min at 37 °C in a shaking bath. At the specified time points, the amount of released AIBI was determined by UV–vis spectroscopy.

### Cell culture

Human OS cells (MNNG/HOS) were purchased from Cell Bank of Shanghai Institute of Biochemistry and Cell Biology, Chinese Academy of Sciences. Bone marrow stromal cells (BMSCs) were kindly provided by Dr. Song Gong (Tongji Medical College, Huazhong University of Science and Technology). The OS cells were maintained in α‐modified essential medium (MEM) (Hyclone) supplemented with 10% fetal bovine serum (FBS) (Gibco; Thermo Fisher Scientific), and 1% penicillin–streptomycin. BMSCs were cultured with Dulbecco’s MEM (DMEM)/ F12 containing 15% FBS and 1% penicillin–streptomycin. All the cells were cultured at 37 °C under an atmosphere of 5% CO2.

### Cellular uptake

To investigate the cellular uptake efficiency of the NPs, MNNG/HOS cells (1 × 10^5^ per well) were seeded and cultured in six-well plates at 37 °C in a humidified atmosphere of 5% CO2 for 24 h. After complete adhesion, the cells were incubated with free ICG and ICG-labeled NPs (ICG@HTP or ICG@HTPR) at an equivalent ICG concentration (15 μg mL^−1^) for 4 h, respectively. For competitive experiment, cells were pretreated with free RGD (2 mg mL^−1^) for 2 h, and then incubated with ICG@HTPR NPs for 4 h. Subsequently, the cells were stained with Hoechst 33,342 (10 μg mL^−1^) for 10 min. After washing with PBS for three times, the cells were imaged by a fluorescence microscope (Olympus Corporation, Tokyo, Japan).

In order to systematically investigate the mechanism of cellular uptake, we then used the MNNG/HOS cells to incubate with ICG@HTPR NPs under different conditions. The seeded MNNG/HOS cells were pre-treated with the clathrin pathway inhibitor chlorpromazine (100 µM), caveolae inhibitor genistein (200 µM), micropinocytosis inhibitor amiloride (50 µM), and phagocytosis inhibitor cytochalasin D (20 µM)) at 37 °C for 1 h and then incubated with ICG@HTPR NPs at 37 °C for 4 h. Cells treated with ICG@HTPR NPs at 37 °C served as the control group. Subsequently, the cells were carefully washed three times with cold PBS and harvested for flow cytometry to determine the fluorescence intensity. Furthermore, internalization assays were also performed at low temperature (4 °C) in the absence of endocytic inhibitors to reflect the energy-dependent endocytosis pathway.

### Mitochondria targeting property

To trace the intracellular localization of AHTPR NPs, MNNG-HOS cells were seed in six-well plates at a density of 1 × 10^5^ per well and incubated for 24. Then the cells were treated with ICG labeled NPs (ICG@HPR and ICG@HTPR) at an equivalent ICG concentration (15 μg mL^−1^) for 6 h. Then, the cells were stained with Mito-Tracker Green (Beyotime Biotechnology, China) for according to the manufacturer’s instruction. Afterwards, the cell nuclei were stained with Hoechst 33,342 (10 μg mL^−1^) for 10 min. After washing with PBS for three times, the mitochondrial co-localization imaging was observed by a fluorescence microscope.

### Intracellular free radical detection

Intracellular generation of free radicals was investigated by a DCFH-DA fluorescent probe. Briefly, MNNG/HOS cells were seed in a six-well plate at a density of 5 × 10^4^/well. After 24 h incubation at 37 °C, PBS, AIBI, HTPR and AHTPR were added. After 4 h incubation for intracellular uptake, the cells were irradiated with 808 nm NIR light at a power density of 1.0 W cm^−2^ for 10 min and incubated for another 4 h. After that, the culture media were discarded and the ROS probe DCF-DA in DMEM (10 µM) were added and incubated for another 45 min. Subsequently, the DMEM media were discarded and followed by PBS rinsing, then observed with a fluorescence microscopy.

### Cell viability assay

The cell viability was determined by using the Cell counting kit-8 (CCK-8) assay. In brief, MNNG/HOS cells (5000 cells per well) were seeded into 96 well plates and cultured for 24 h to allow cell attachment. After the corresponding treatment, the cells were cultured for another 24 h at 37 °C. Afterwards, the supernatant was discarded and washed twice carefully with PBS. Then, 100 μL fresh medium containing 10% CCK-8 solution were added. After incubation in dark for 2 h, the cell viability was assessed by measuring sample absorbance at 450 nm by a microplate reader (Biotek, Winooski, VT, USA).

### Live/dead cells staining

The live/dead cell staining assay was utilized to intuitively evaluate the cell apoptosis-inducing activity of different formulations in MNNG/HOS cells. Briefly, cells were seeded in 96-well plates at a density of 5000 cells per well. After 24 h of incubation, the cells were treated according to the description aforementioned for another 24 h. Afterwards, the cells were stained with 2 μM Calcein-AM and 2 μM propidium iodide (PI) for 15 min. Following staining, cells were washed twice with PBS and observed by using a fluorescence microscopy.

### Cell apoptosis

MNNG/HOS cells were seeded in six-well plates at a density of 1 × 10^5^ per well. After incubation at 37 °C for 24 h, the cells were treated according to the description aforementioned. Afterwards, the cell apoptosis was assessed by flow cytometry (Becton Dickinson, Franklin Lakes, New Jersey, USA) after cells were stained with Annexin V- fluorescein isothiocyanate (FITC) / propidium iodide (PI) dual staining (Nanjing Keygen Biotech, Nanjing, China) according to the manufacturer’s protocol.

### Assessment of intracellular GSH

The intracellular GSH was detected using Ellman’s reagents. In Brief, MNNG/HOS cells were incubated with different materials and treated accordingly. Then the treated cells were collected by repeated cycle of freezing and thawing and then centrifuged to collect the supernatants for the measurement of GSH based on the standard protocol.

### Analysis of mitochondrial membrane potential (MMP) and intracellular ATP levels

The change of mitochondrial membrane potential was determined by 5,5′,6,6′-tetrachloro-1,1′,3,3′-tetraethylbenzimidazolocarbocyanine iodide (JC-1) detection Kit. MNNG/HOS cells were seeded in six-well plates and cultured for 24 h. After incubation with PBS, AIBI, AHPR and AHTPR NPs for 6 h, the cells were exposed to an NIR laser (808 nm, 1.0 W cm^−2^) for 10 min. Afterwards, the cells were stained by JC-1 (5 μg mL^−1^) for 20 min. After washing with PBS, the cells were imaged by fluorescence microscopy.

The intracellular adenosine triphosphate (ATP) levels were detected by ATP determination kit (Beyotime, China). Briefly, the cells were collected and lysed with lysis buffer after the corresponding treatment for 24 h. After centrifugation at 12,000 rpm at 4 °C for 5 min, the supernatant was harvested for subsequent determination. The ATP concentration of the samples was determined by a standard curve which was generated according to the manufacturer’s protocol.

### Western blotting analysis

After different treatments, the MNNG/HOS cells were lysed by RIPA buffer (Thermo Fisher Scientific) containing protease inhibitors and phosphatase inhibitors and the concentration of protein was determined via the BCA Protein Assay kit (Beyotime Biotechnology Co. Ltd). Subsequently, the same protein concentrations of various samples were separated by 12% of sodium dodecyl-polyacrylamide gel electrophoresis (SDS-PAGE) and blotted on polyvinylidene difluoride (PVDF) membranes. After being blocked by 5% non-fat milk for 1 h, the PVDF membranes were incubated with primary antibody overnight at 4 °C. Then the PVDF membranes were washed with TBST for three times and incubated with the secondary antibody for 1 h at room temperature. Afterwards, the membranes were washed three times with TBST buffer and detected by using electrochemiluminescence detection reagent (EMD Millipore) according to the manufacturer’s instructions.

### Animal models

Female BALB/c nude mice (4–6 weeks) were purchased from Beijing HFK Bioscience Co. Ltd. All animal experiments were performed according to protocols approved by the Institutional Animal Care and Use Committee (IACUC) at Tongji Medical College, Huazhong University of Science and Technology (IACUC Number: S2503).

To establish tumor models, MNNG/HOS cells (1 × 10^7^) suspended in 200 μL cold PBS were subcutaneously injected into the right flank of nude mice. The tumor-bearing mice were treated when the tumor volume reached around 100 mm^3^. The tumor volume was calculated using the formula: Tumor volume = 1/2 × length × (width)^2^. Where the length and width determined by vernier caliper are the longest and shortest dimension of the tumor, respectively.

### In vivo fluorescence imaging and photothermal imaging

For vivo tumor fluorescence imaging, and 200 µL of ICG labeled NPs (ICG@HTP and ICG@HTPR, 2 mg mL^−1^) was injected into the tumor-bearing mice via tail vein. The mice (n = 3) were anesthetized and image using IVIS small animal imaging system (PerkinElmer Inc., Waltham, USA) at the appointed time points (0 h, 1 h, 3 h, 6 h, 12 h and 24 h). After injection for 24 h, the mice were sacrificed, and the tumors and main organs (heart, lung, liver, spleen, and kidney) were harvested for ex vivo imaging.

Photothermal imaging was performed following a similar procedure. Briefly, 200 μL PBS or NPs (AHTP and AHTPR, 2 mg mL^−1^) were intravenously injected into MNNG/HOS tumor-bearing mice (n = 3), respectively. The tumor regions of the mice were irradiated with an 808 nm laser (1.0 W cm^−2^) continuously for 5 min after 6 h post-injection. The changes in temperature in the tumor region were measured by infrared thermal imaging camera (Testo 865, Testo, Schwarzwald, Germany) every minute.

### In vivo synergistic cancer therapy and biosafety

When the volume of tumors reached approximately 100 mm^3^, the MNNG/HOS tumor-bearing mice were randomly assigned to five groups (n = 5): (1) control group, (2) NIR, (3) AIBI + NIR, (4) AHTP + NIR, and (5) AHTPR + NIR. The mice without any treatment served as control group. 200 µL of different materials (10 mg kg^−1^) were injected into the tail vein of tumor-bearing mice every 2 d. At 6 h post-injection, the tumor site was treated with 808 nm NIR laser irradiation (1.0 W cm^−2^, 5 min). The tumor volume and body weight were monitored every other day during the therapy. All mice were sacrificed after the therapy for two weeks, the tumors were extracted, weighed and photographed. Thereafter, the tumor tissues were collected and examined by hematoxylin and eosin (H&E) staining, Ki-67 and terminal deoxynucleotidyl transferase-mediated dUTP nick end labeling (TUNEL) assay. To assess the in vivo biosafety of the treatments, major organs (heart, liver, spleen, lung, and kidney) were extracted for H&E staining assay. Furthermore, the blood of the mice was extracted to perform the hematology analysis.

### Statistical analysis

All the experiments were performed three times and the data were presented as mean ± standard deviation (SD). The statistical significance was determined by Student’s t-test and one-way analysis of variance (ANOVA) by using GraphPad Prism version 7.00. P < 0.05 was considered as statistically significant.

## Results and discussion

### Preparation and characterization of the AIBI@H-mMnO_2_-TPP@PDA-RGD (AHTPR)

The procedure for the synthesis of the NPs was illustrated in Scheme 1. In brief, H-mMnO_2_ NPs were first fabricated according to a previously reported protocol with slight modifications [23]. For the preparation of TPP modified H-mMnO_2_ (H-mMnO_2_-TPP), the surface of H-mMnO_2_ was functionalized with amine groups. Subsequently, AIBI was loaded into H-mMnO_2_-TPP NPs to obtain AIBI@H-mMnO_2_-TPP (AHT). PDA was then coated on the surface of AHT as gatekeepers to prevent the premature leakage of cargo to obtain AIBI@H-mMnO2-TPP@PDA (AHTP). Finally, the active-targeting ligand RGD was conjugated to the PDA coating through Michael addition reaction [33], resulting in AIBI@H-mMnO_2_-TPP@PDA-RGD (AHTPR).

**Fig. 1:**
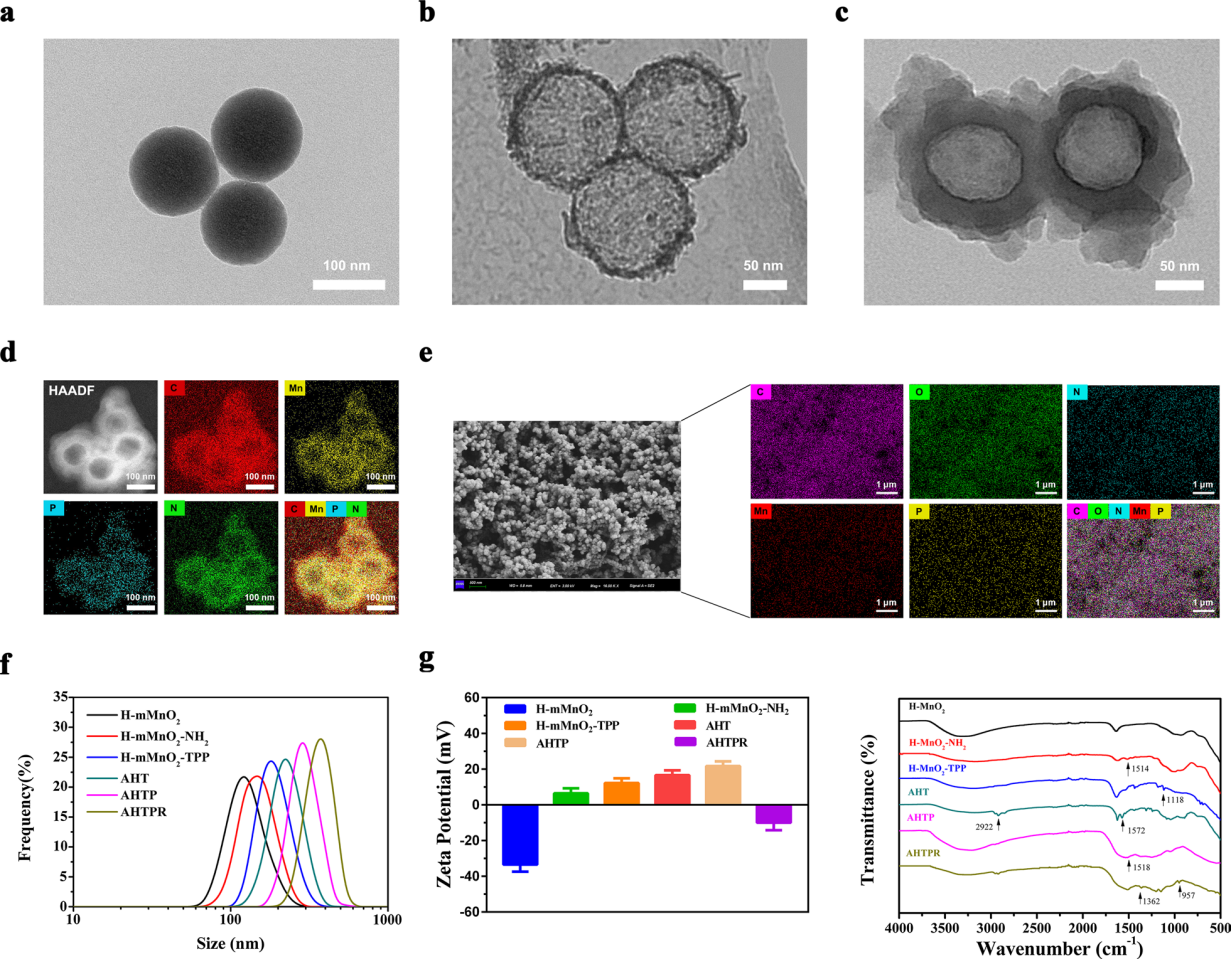
The characterization analysis of the synthesized formulations. TEM images of **a** sSiO_2_, **b** sSiO_2_@MnO_2_, and **c** AHTPR. **d**, **e** TEM mapping and SEM mapping of the AHTPR NPs. **f** The DLS size and **g** Zeta potential, and **h** FTIR spectra of H-mMnO_2_, H-mMnO_2_-NH_2_, H-mMnO_2_-TPP, HTP, AHTP, and AHTPR

The morphology and size of the NPs were characterized by a TEM. As depicted in Fig. 1a and Additional file 1: Fig. S1, the as-prepare sSiO_2_ NPs displayed uniform sphere-like morphology and the average size was approximately 120.5 nm as determined by TEM. Obviously, the TEM image of H-MnO_2_ informed the hollow mesoporous structure (Fig. 1b). Furthermore, the Brunauer–Emmett–Teller (BET) approach was used to investigate the surface area and pore size distributions of the samples. As shown in Additional file 1: Fig. S2 the BET specific surface area of the H-mMnO_2_-TPP NPs was determined to be 285.65 m^2^ g^−1^ with the average pore size distribution at around 3.39 nm. These results informed that the as-prepared H-mMnO2 NPs possessed potential as ideal nanocarrier for delivering therapeutic agents. As revealed in Fig. 1c, the obtained AHTPR NPs obviously revealed a distinct two-layer structure. Further, the TEM mapping confirmed the core–shell structure of the AHTPR NPs (Fig. 1d). Additionally, SEM mapping (Fig. 1e) and the corresponding energy-dispersive X-ray spectroscopy (EDS) (Additional file 1: Fig. S3) indicated the coexistence of C, N, O, Mn, and P elements. The size distribution of the NPs was measured by DLS analysis. As displayed in Fig. 1f, the hydrodynamic diameters of H-mMnO_2_, H-mMnO_2_-NH_2_, H-mMnO_2_-TPP, AHT, AHTP, and AHTPR NPs were about 128.8 nm, 151.0 nm, and 191.3 nm, 229.4 nm, 295.6 nm, and 370.0 nm respectively. Notably, the size measured by DLS was larger than the result obtained from TEM images, which might be ascribed to the hydration of NPs in water [12].

The dispersity of the final samples (AHTPR NPs) was monitored in different media (water, α-MEM culture medium with or without 10% FBS) over a prolonged incubation time up to 7 days. As shown in Additional file 1: Fig. S4a, AHTPR NPs could be well dispersed in water, α-MEM culture medium with or without 10% FBS with no obvious agglomeration or precipitation after being stored for one week, which is conducive to prolonging blood circulation time in vivo for use. Subsequently, the colloidal stability of AHTPR NPs was determined by characterizing the hydrodynamic diameters. As depicted in Additional file 1: Fig. S4b, noticeable changes were found in hydrodynamic diameters of the as-prepared AHTPR NPs during 7 d of incubation in water, α-MEM culture medium with or without 10% FBS, illustrating their remarkable long-term stability in physiological solution, which makes possible for their prolonged circulation in vivo.

**Fig. 2:**
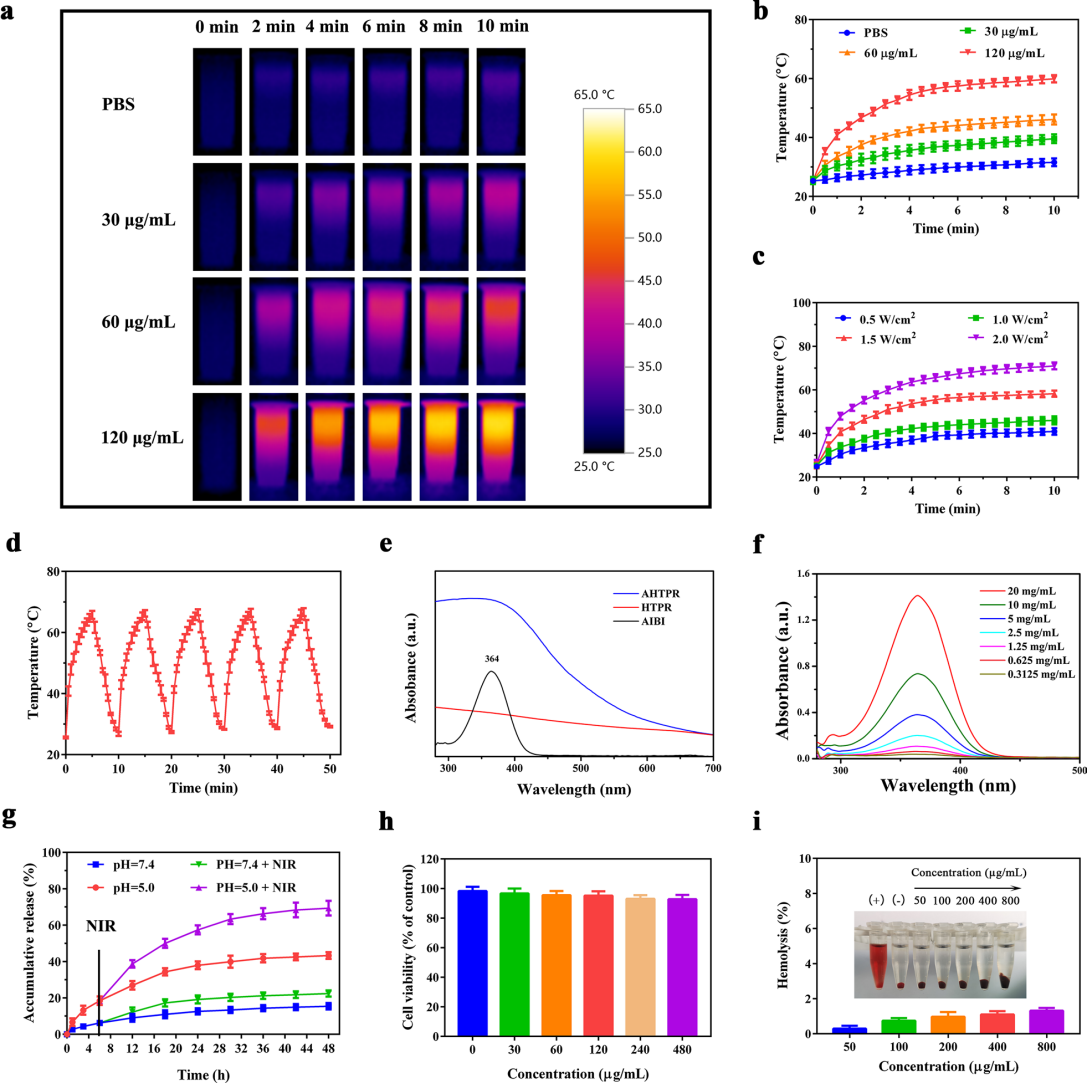
Photothermal properties, release profiles and biocompatibility of AHTPR NPs in vitro. **a**, **b** The infrared thermal images and time-dependent temperature elevation of AHTPR at various concentrations under 1.0 W cm^−2^ 808 nm laser irradiation. **c** Time-dependent temperature change of AHTPR (120 µg mL^−1^) under 808 nm laser irradiation with different laser power. **d** Photothermal stability curve of AHTPR (120 µg mL^−1^) NPs following on/off NIR laser irradiation (2.0 W cm^−2^) for 5 cycles. **e** UV–vis spectra of AIBI, HTPR and AHTPR. **f** The UV–vis absorption spectra of AIBI with different concentrations. **g** The release profiles of AIBI from AHTPR in PBS with different pH values without or with NIR irradiation (808 nm, 1.0 W cm^−2^, 10 min). **h** Cell viability of BMSCs after 24 h of treatments with various concentrations of AHTPR NPs. **i** Hemolysis assay of different concentrations of AHTPR samples

The zeta potential changes during the modification process were monitored. As shown in Fig. 2g, the zeta potentials of the H-mMnO_2_, H-mMnO_2_-NH_2_, H-mMnO_2_-TPP, AHT, AHTP, and AHTPR NPs were − 33.45 ± 4.01 mv, 6.42 ± 2.78 mv, 12.16 ± 2.69 mv, 16.60 ± 2.74 mv, 21.68 ± 2.66 mv, and − 9.97 ± 4.27 mv, respectively. These step-wise altered zeta potentials indicated the successful construction of the nano-system. Furthermore, FTIR spectroscopy was performed to validate the surface chemistry changes along the modification process. As revealed in Fig. 1h, the bending vibration of -NH_2_ at 1514 cm^−1^ confirmed the successful modification of amino group on H-mMnO_2_. Compared with the spectra of H-mMnO_2_-NH_2_, a new peak at 1118 cm^−1^ was observed in H-mMnO_2_-TPP, which was consistent with the C-N stretching of amide groups [34]. After loading AIBI, the AHT NPs demonstrated two characteristic peaks, such as the –CH_3_ stretching vibration peak at 2921 cm^−1^ and the N=N stretching vibration peak at 1572 cm^−1^, which confirmed the AIBI were successfully loaded into the AHT NPs. In comparison with AHT, the peaks at 1518 cm^−1^ in the spectrum of AHTP might be were ascribed to the aromatic C = C stretching vibration of indole or indoline structures [35], which indicated the PDA-coating was successfully conjugated on the surface of the AHT NPs. Furthermore, the emergence of absorbance peaks at 1362 cm^−1^ and 957 cm^−1^ in the spectrum of AHTPR, ascribing to glycine (− CH_2_C=O) and aspartic acid (COOH) of the RGD peptide, respectively [36]. Based on these characterization results, we concluded that the AHTPR NPs were successfully synthesized.

### Photothermal properties of NPs

To explore the photothermal performance of the as-synthesized AHTPR NPs, the temperature changes of different preparations after exposure to NIR irradiation (808 nm) were recorded by an infrared thermal imaging camera. As exhibited in Additional file 1: Fig. S5, in comparison with other formulations (PBS and H-mMnO_2_), the temperature of HTPR (at equal H-mMnO_2_ concentration, 120 μg mL^−1^) NPs exhibited prominent photothermal conversion efficiency under the same condition, which might be contributed to the combination of two excellent photothermal agents (PDA and H-mMnO_2_) [23]. Subsequently, the photothermal-conversion performance of AHTPR NPs at various concentrations under NIR laser irradiation (1.0 W cm^−2^, 10 min) was investigated. As shown in Fig. 2a and b, the temperature of AHTPR NPs exhibited an obvious concentration- and time-dependent rise. In marked contrast, the temperature of PBS rose slightly under the same conditions. Furthermore, it could be found that the photothermal efficiency of the AHTPR NPs displayed a promising laser power intensity-dependent manner (Fig. 2c). To validate the photothermal stability of the NPs, AHTPR NPs were challenged with five times heating–cooling cycles. As displayed in Fig. 2d, no obvious change was found in the temperature variation curves after laser irradiation for five cycles. The photothermal conversion efficiency of the NPs was calculated according to the previous method [37, 38]. According to the temperature change plot curve before and after irradiation (Additional file 1: Fig. S6a, b), the photothermal conversion efficiency (η) of the AHTPR NPs was calculated by the formula as 37.61%. Collectively, the prepared AHTPR NPs exhibited prominent photothermal conversion ability and outstanding photothermal stability, demonstrating their remarkable potential application value as efficient thermal agents for anticancer therapy.

### Drug loading and release

In order to prevent premature drug leakage during circulation in the blood, HTPR was employed as a controlled drug delivery carrier for AIBI. As shown in Fig. 2e, AHTPR exhibited the characteristic absorbance peaks of AIBI at 364 nm as expected, indicating that AIBI was successfully loaded into HTPR. According to the absorption intensity of different concentrations of AIBI at 364 nm (Fig. 2f), the standard curve of AIBI was established (Additional file 1: Fig. S7). Subsequently, the loading efficiency of AIBI was calculated to be around 52.36 ± 3.13% and the corresponding encapsulation efficiency was about 27.63 ± 3.54%.

To investigate the gating effect of the PDA shell for controlled release of AIBI, the release behavior of the NPs under different pH conditions was first evaluated. As shown in Fig. 2g, only slow release of AIBI (15.4%) from NPs was observed at PH 7.4. Nevertheless, the amount of released AIBI increased up to 43.3% at pH 5.5 within the same incubation time of 48 h, suggesting that the AIBI release from the NPs was significantly pH-dependent. That might be attributed to the depolymerization of PDA shell in the acidic environment. The bare release of agents at neutral physiological environment indicated AHTPR could keep safe for normal tissue. Subsequently, the NIR-triggered release behavior of the NPs at PH 5.0 or 7.4 was evaluated under 808 nm laser irradiation. As illustrated in Fig. 2g, the AIBI release was dramatically increased at pH 5.0 after NIR irradiation (1.0 W cm^−2^) for 10 min, conferring a cumulative release amount of 69.3%. In contrast, the release rates of AIBI were slightly increased at pH 7.4 under the same conditions. Consequently, the above finding confirmed that the pH and NIR-laser irradiation could act as an intelligent switch for the nanocomposites to control the release of AIBI, which could prevent drug leakage and reduce adverse effects to normal tissues.

### Biocompatibility of the NPs

The biocompatibility of NPs is of great importance for their systemic administration as drug delivery carriers prior to their clinical application. The cytotoxicity of AHTPR to normal cells was firstly evaluated on BMSCs by using CCK8 assay, and no significant cytotoxicity could be observed even at the concentration of 480 µg mL^−1^ (Fig. 2h), proving the excellent biocompatibility of the materials. In addition, the hemolysis assay was also performed to assess the hemocompatibility of the NPs. As displayed in Fig. 2i, there was negligible hemolysis of RBCs in the PBS and AHTPR groups, even at the highest testing concentration (800 μg mL^−1^). In contrast, the color of the positive control (pure water) turned red because the RBCs were broken to release the hemoglobin. Collectively, the prepared AHTPR NPs showed excellent biocompatibility and hemocompatibility, making the AHTPR an outstanding candidate for future exploration.

### Cellular uptake and mitochondrial targeting effects

**Fig. 3:**
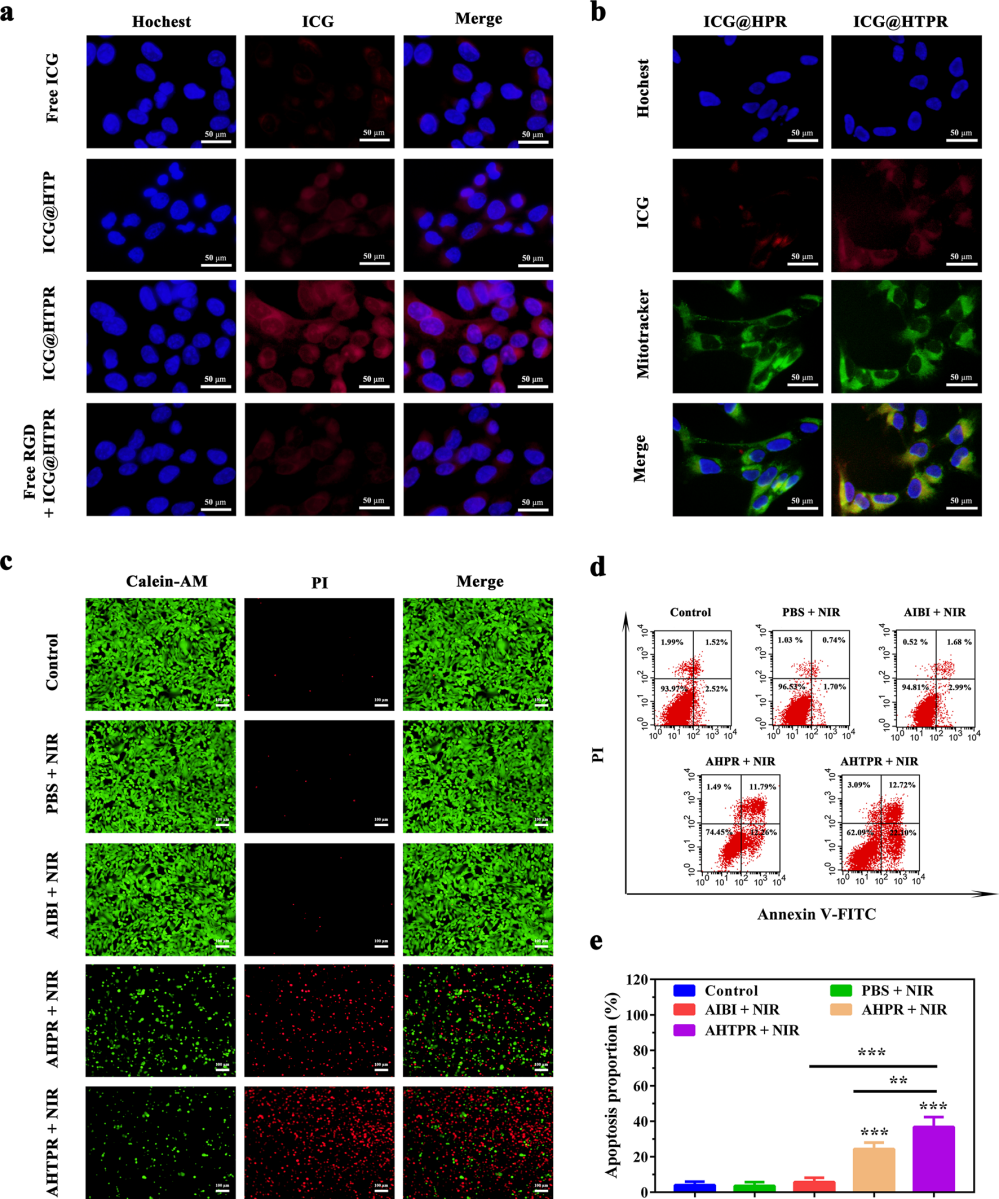
In Vitro Cellular Assay. **a** Fluorescence microscopy images of MNNG/HOS cells after 4 h incubation with ICG, ICG@HTP and ICG@HTPR NPs. **b** Co-localization of the test NPs into mitochondria of MNNG/HOS cells. **c** Corresponding fluorescence microscopy images of MNNG/HOS cells stained with Calcein-AM and PI. **d** Flow cytometry apoptosis experiment based on annexin V-FITC/PI staining of MNNG/HOS cells after incubation with different formulations for 24 h, including control group, PBS, AIBI (50 μg/mL), AHPR (120 μg/mL, equivalent AIBI dosage of 50 µg/mL), AHTPR (120 μg/mL, equivalent AIBI dosage of 50 µg/mL), and 808 nm laser (1.0 W cm^−2^, 5 min). Cells without treatment were used as control. **e** Apoptosis ratios of cells after corresponding treatment was quantified. (*P < 0.05, **P < 0.01, ***P < 0.001)

It is essential to effective targeted delivery the therapy agents into tumors for nanomaterial-based drug-delivery systems [39]. To study the cellular uptake behavior of the NPs, the cellular uptake of free ICG and ICG-labeled NPs were determined by a fluorescence microscope. As shown in Fig. 3a, the red fluorescence intensity of ICG in MNNG/HOS cells treated with ICG@HTPR NPs was significantly stronger than that of the cells treated with ICG@HTP NPs and free ICG. Enhanced cellular uptake of the ICG@HTPR NPs might be ascribed to the capacity of the RGD peptide which can attach integrin ανβ3 integrin overexpressed in OS cells. Additionally, the competitive experiment demonstrated that the intracellular fluorescence signals were markedly decreased when pre-treated with free RGD peptide (Fig. 3a), confirming that endocytosis of the RGD-modified NPs was partly ascribed to integrin receptor-mediated endocytosis pathway. Taken together, these results demonstrated that our designed RGD-functionalized nanocarriers could be efficiently internalized by MNNG/HOS cells, which provided the potential for tumor-specific drug delivery.

To further investigate the mechanism of cellular uptake, we then used the MNNG/HOS cells to incubate with ICG@HTPR NPs under different conditions. Since the uptake of nano-formulations was largely mediated through clathrin-mediated endocytosis, caveolae-mediated endocytosis, macropinocytosis, and phagocytosis [40, 41], the MNNG/HOS cells were pre-treated with different endocytosis inhibitors: the clathrin pathway inhibitor chlorpromazine, caveolae inhibitor genistein, micropinocytosis inhibitor amiloride, and phagocytosis inhibitor cytochalasin D. As shown in Additional file 1: Fig. S8, the cellular uptake of ICG@HTPR by MNNG/HOS cells was obviously suppressed by the inhibitors of amiloride and chlorpromazine, but was not affected by cytochalasin D and genistein treatment. The results suggested that the cellular uptake of the NPs was mainly depends on macropinocytosis- and clathrin-mediated endocytosis pathways. Furthermore, internalization assays were also performed at low temperature (4 °C) in the absence of endocytic inhibitors to reflect the energy-dependent endocytosis pathway. As displayed in Additional file 1: Fig. S8, the cell uptake efficiency of ICG@HTPR was more enhanced at 37 °C than at 4 °C in MNNG/HOS cells. This indicated that the uptake of NPs in MNNG/HOS cells was mainly an energy-dependent process.

According to previous studies [14, 42], ICG@HTPR NPs were expected to be localized the mitochondria due to the TPP targeting ability. The co-localization of the NPs into mitochondria was evaluated by a fluorescence microscope. As revealed in Fig. 3b, a significant red fluorescence was observed in ICG@HTPR NPs group, which was stronger than that in ICG@HPR groups, and it nearly overlapped with the green fluorescence of mitochondria. This result confirmed that the TPP functionalized NPs exhibited excellent mitochondria-targeting ability, which also demonstrated that the PDA shell could be removed to expose TPP in the acidic environment before reaching the mitochondria.

### In vitro cell killing evaluation

Encourage by the excellent mitochondrial targeting ability of the NPs, the synergistic therapeutic effects of the engineered platform were assessed in vitro. Firstly, live/dead cell staining assay was utilized to directly visualize cell death after receiving various treatments under a fluorescence microscope. As displayed in Fig. 3c, most cells in PBS + NIR group were alive as evidenced by the strong green fluorescence and undetectable red fluorescence signal, suggesting that low-temperature laser irradiation alone showed little cytotoxicity. Similarly, cells treated with free AIBI displayed almost undetectable red fluorescence signal under low-temperature laser irradiation. Although increased cells with red fluorescence were presented in the AHPR with irradiation treated group, more cells were killed in AHTPR coupled with NIR irradiation treated group were dead, confirming that the therapeutic efficacy could be improved with mitochondria-targeted treatment.

In addition, the in vitro anticancer activity was quantitatively evaluated in MNNG/HOS cells by CCK8 assay. It was found that AHTPR induced the most severe cytotoxicity to MNNG/HOS cells. Only 20.0% cells kept alive after being treated with AHTPR + NIR for 24 h, while there were 93.1% or 43.5% of viable cells for those treated with AIBI + NIR or AHPR + NIR, respectively (Additional file 1: Fig. S9). Subsequently, we further verified the apoptosis of MNNG/HOS cells after various treatments with flow cytometry using annexin V-FITC/PI staining. Consistently, we found that AHTPR + NIR could induce a dramatically higher level of cell apoptosis (Fig. 3d, e). Collectively, these results confirmed the excellent synergistic therapeutic effect of mitochondria-targeting LPTT and TDT.

### Accumulation of free radicals in mitochondria induced oxidative damage

**Fig. 4:**
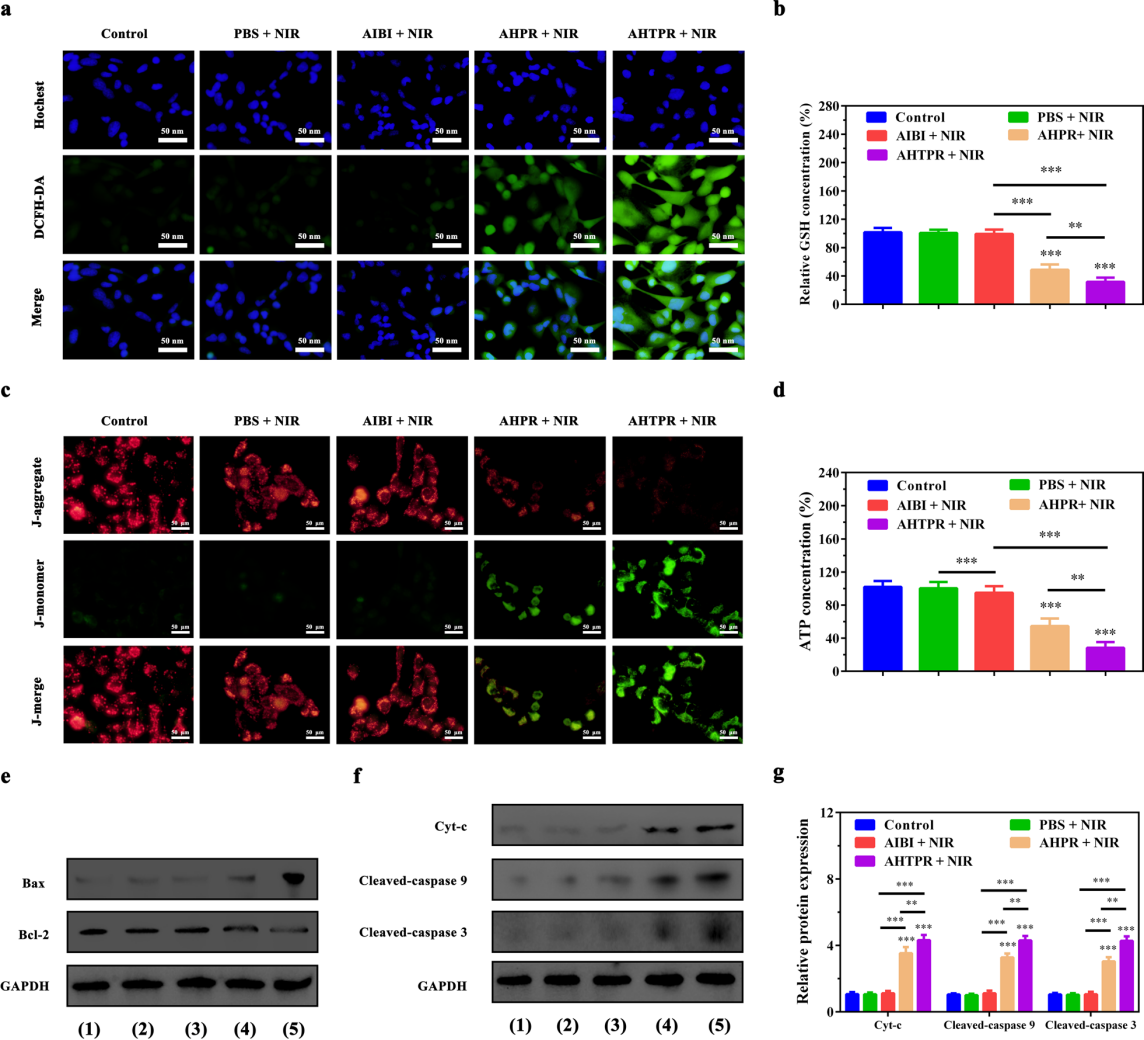
Accumulation of free radicals in mitochondria induced oxidative damage. **a** The generation of alkyl radicals in MNNG/HOS cells after corresponding treatment. **b** The relative level of GSH of MNNG/HOS cells after corresponding treatment. **c** Detection of mitochondrial membrane potential (ΔΨm). **d** Intracellular ATP levels of MNNG/HOS cells after corresponding treatment. **e** The effect of various treatment on the expression of Bax and Bcl-2 in MNNG/HOS cells. (Groups included (1) control, (2) PBS + NIR, (3) AIBI + NIR, (4) AHPR + NIR, (5) AHTPR + NIR). **f** The effect of various treatment on the expression of Cyt-c, cleaved-caspase 9 and cleaved-caspase 3 in MNNG/HOS cells. **g** Relative protein levels of Cyt-c, cleaved-caspase 9 and cleaved-caspase 3 in MNNG/HOS cells (*P < 0.05, **P < 0.01, ***P < 0.001)

AIBI, one of azo initiators, could be decomposed rapidly under heat stimulation to produce alkyl radicals for killing cancer cells. ESR analysis was performed to evaluate the generation of free radical in vitro. The 5,5′-dimethylpyrroline-1-oxide (DMPO) was used as a spin trap agent to capture the released radicals. As shown in Additional file 1: Fig. S10, a group of typical radical peaks appeared in the solution containing AHTPR after irradiating with the 808 nm laser irradiation for 5 min. By contrast, no alkyl radical peaks were observed in the solution containing AIBI or HTPR under the same condition, suggesting that the capability of AHTPR NPs for oxygen-irrelevant free-radical generation upon laser irradiation. Subsequently, to evaluate the intracellular free-radical levels, DCFH-DA was employed to as a fluorescent probe to verify the generated capacity of intracellular free radicals. As presented in Fig. 4a and Additional file 1: Fig. S11, no green fluorescence was observed for the control group with NIR laser alone. Notably, cells treated with AHTPR + NIR exhibited distinct green fluorescence compared with other treatments under the same conditions. Such findings indicated that the precise subcellular localization of nanocomposites could dramatically enhance the free-radicals generation at a low temperature (< 45 °C). More importantly, it is well known that MnO_2_-based nanoplatform could effectively remit the effect of excessive GSH on counteracting the damaging effects of alkyl radicals [12, 19, 43]. To confirm this, the degradation of the as-prepared H-mMnO_2_ NPs in the presence of GSH was investigated. First, the morphologic changes of H-mMnO_2_ NPs at different GSH concentrations (0, 1, and 2 mM, respectively) were observed by TEM. As shown in Additional file 1: Fig. S12, the hollow structure of MnO_2_ was almost decomposed when treated GSH with a concentration of 2 mM, proving the GSH-responsive degradation of H-mMnO_2_. Subsequently, X-ray photoelectron spectroscopy (XPS) was used to determine the valence state of the NPs in the presence of 2 mM GSH or not. As displayed in Additional file 1: Fig. S13, two peaks at 641.9 and 653.6 eV ascribed to Mn 2p_3/2_ and Mn 2p_1/2_, respectively, which demonstrated the elemental valence of Mn in the synthesized H-mMnO_2_ was IV [37]. After the treatment with GSH, two major peaks for Mn 2p_3/2_ and Mn 2p_1/2_ were present at 641.5 and 653.2 eV [44, 45] (Additional file 1: Fig. S13b), respectively, which indicated the presence of Mn^2+^ valence state. From the XPS analysis, it could be seen that the elemental valence of Mn in the H-mMnO_2_ NPs changed from IV to II after treatment with GSH.

The GSH levels of MNNG/HOS cells after various treatment were then assessed using a GSH assay kit. As predicted, a dramatic reduction in intracellular GSH level was observed for the AHTPR + NIR group (Fig. 4b). These results illustrated that AHTPR could significantly enhance the accumulation of oxygen-irrelevant free radicals in mitochondria through the targeted release of free radicals in mitochondria and MnO_2_-mediated GSH depletion.

Since mitochondria are extremely sensitive to free-radical mediated damages, selectively accumulation of free radicals in mitochondria would trigger mitochondrial dysfunction, decrease the mitochondrial membrane potential, and finally induce tumor cell apoptosis [14, 15]. The mitochondrial membrane potential (ΔΨm) changes after various treatments were firstly examined by performing JC-1 assays. As shown in Fig. 4c, the weakest red fluorescence was detected in AHTPR + NIR group compared with other treatments, clearly indicating a dramatic decrease in the mitochondrial membrane potential and the destruction of membrane integrity.

Mitochondria are recognized as the pivotal sources of ATP production to meet the energy demands of the cell [46]. Accumulating evidence has revealed that mitochondrial dysfunction would result in defective oxidative respiratory chain, which might decrease intracellular ATP levels and could not provide the required energy for tumor growth [47, 48]. Subsequently, we continued to investigate if the synergistic therapeutic effect could influence the ATP production in MNNG/HOS cells. As shown in Fig. 4d, compared with other groups, the intracellular ATP concentration decreased tremendously in AHTPR + NIR treated cells. These results indicated that mitochondria-targeting LPTT and TDT could not only disrupt mitochondrial membrane potential but also inhibit the cellular ATP production, ultimately inducing the mitochondria-mediated cell apoptosis.

**Fig. 5:**
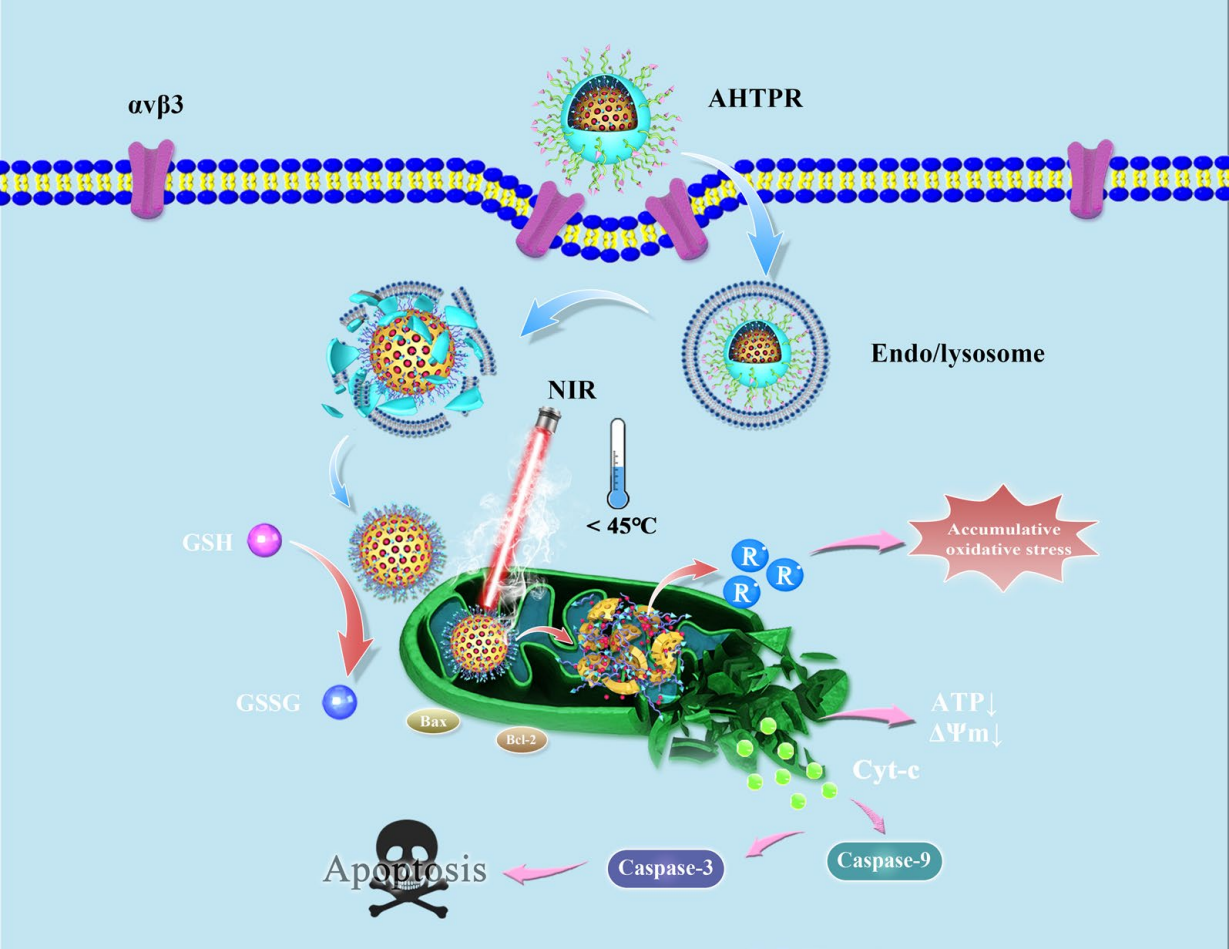
Schematic illustration of the underlying mechanism for the synergistic mitochondria-targeted LPTT/TDT

To further illustrate the mitochondrial mediated apoptosis pathway, the expression of apoptosis‐related proteins was investigated in MNNG/HOS cells by western-blot analysis. As revealed by western blotting (Fig. 4e) and relative quantitative analysis (Additional file 1: Fig. S14), the cells treated with AHTPR + NIR significantly upregulated the proapoptotic protein Bax and dramatically down-regulated the anti-apoptotic protein Bcl-2. Meanwhile, the expression of apoptosis protein markers (Cyt-c, cleaved-caspase-9 and cleaved-caspase-3) were markedly increased in AHTPR + NIR group, compared with other three groups (Fig. 4f, g). Taken together, all these results above revealed that AHTPR coupled with low-temperature laser irradiation could effectively decrease mitochondrial membrane potential, reduce ATP production, and ultimately inducing mitochondrial associated apoptosis in cancer cells (Fig. 5), exhibiting the advantages of mitochondria-targeted LPTT/TDT.

### Biodistribution and photothermal effect in vivo

**Fig. 6:**
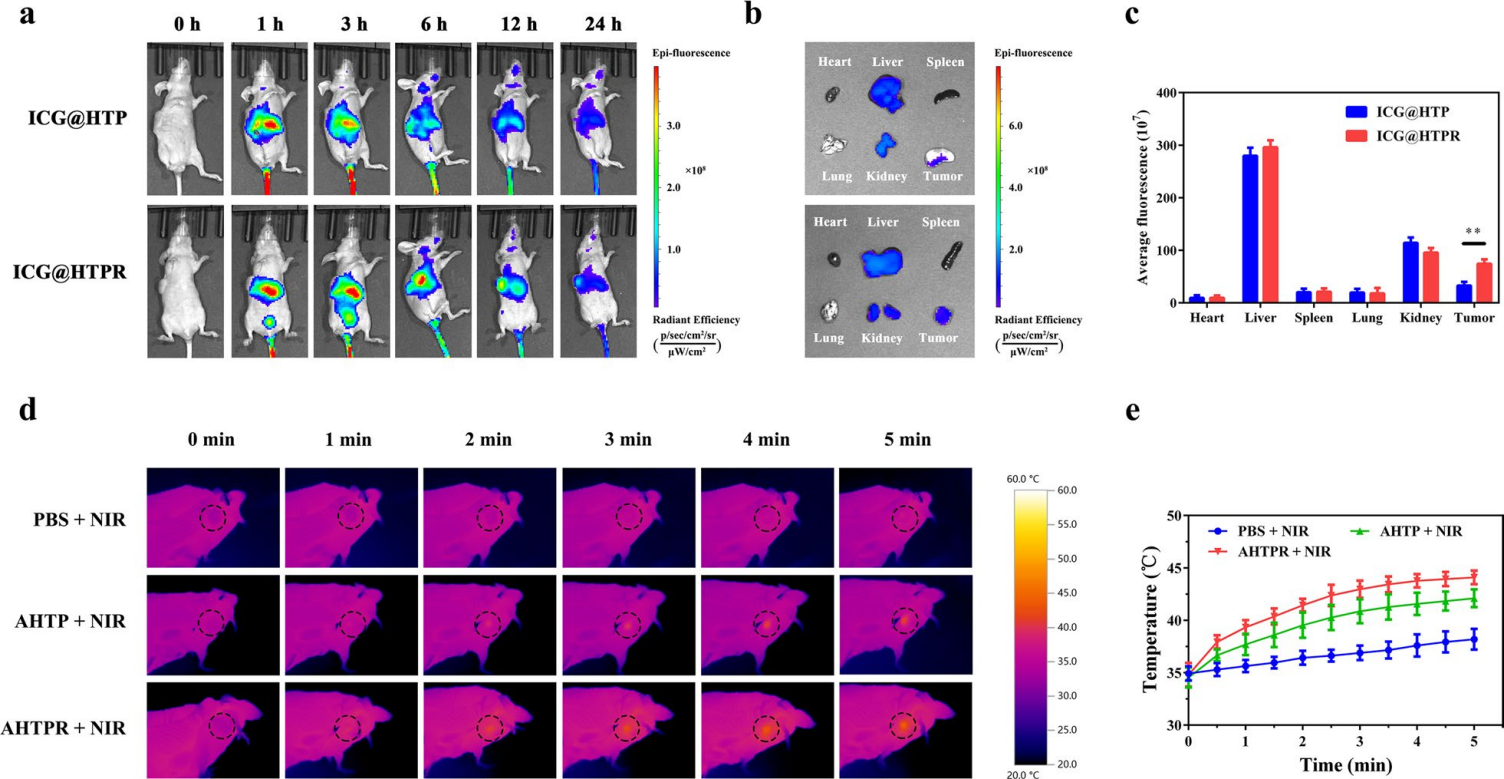
Biodistribution and photothermal effect of the HTPR NPs in vivo. **a** In vivo fluorescence images of MNNG/HOS tumor-bearing mice at indicated time point after the intravenous injection with ICG labeled NPs (ICG@HTP and ICG@HTPR). **b** Ex vivo fluorescence images of tumor and main organs after 24 h of post-injection. **c** Semiquantitative analysis of fluorescence intensity in the tumors and major organs. **d**, **e** In vivo thermal images and temperature variation curves of tumor region recorded by using the NIR thermal camera during NIR laser irradiation (1.0 W cm^−2^)

To investigate the in vivo biodistribution of the nanocomposites, MNNG/HOS tumor-bearing mouse model was established for in vivo imaging using a small animal imaging system. As shown in Fig. 6a, the accumulated fluorescence was observed at various time intervals, demonstrating that the NPs could gradually accumulate in the tumors. The intensity of fluorescence within the tumor tissues reached a maximum after 6 h post-injection and decreased with the prolonged time. After 24 h post-injection, the tumor site still maintained the relative strong fluorescence, indicating the long retention of the NPs. Meanwhile, the tumor tissues as well as major organs (heart, liver spleen, lung and kidney) were harvested for ex vivo imaging. Notably, the fluorescence intensity in the tumor tissues was still much stronger than in the major organs by semiquantitative mean fluorescence intensity analysis (Fig. 6b, c), further confirming the superior enrichment capacity of the RGD modified NPs in tumor. The above results exhibited that the ICG@HTPR NPs could prominently accumulated in tumor region, which was owing to enhanced permeability and retention (EPR) effect-mediated passive and RGD-induced active tumor-targeting delivery.

Based on the above in vivo imaging observation, we selected 6 h post-injection as the optimal timepoint for laser irradiation. As indicated in Fig. 6d and e, the temperature in the free PBS treated mice increased slightly after 5 min of NIR laser irradiation. Notably, the temperature of tumor site in the AHTPR group could reach about 45 °C under the same condition, which is enough to lead the decomposition of AIBI and the release of free radicals for therapy. These results highlighted the promising photothermal performance of AHTPR in vivo.

### In vivo antitumor study

**Fig. 7:**
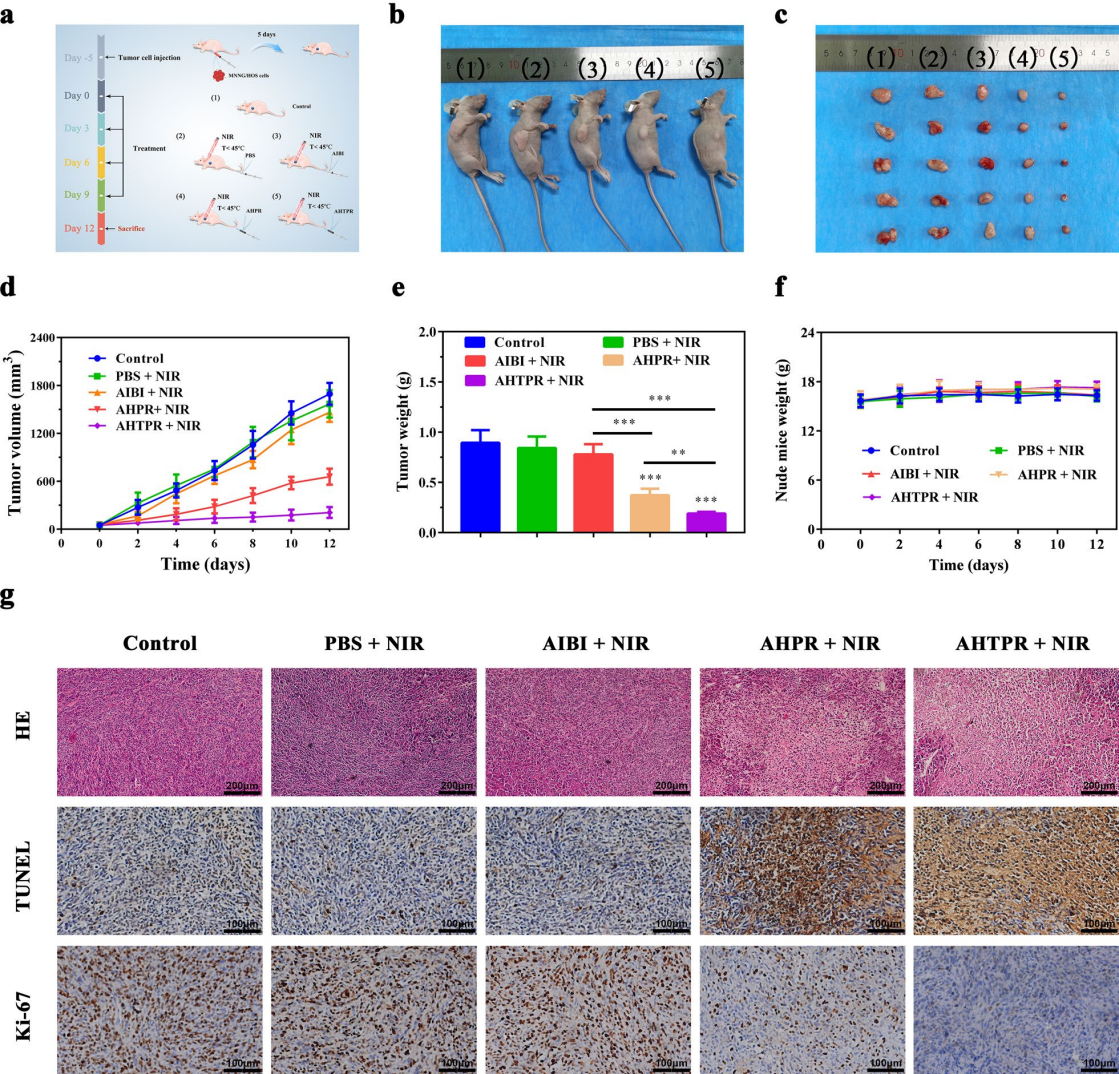
Evaluation of the synergistic therapeutic efficacy of formulations in vivo. **a** Schematic diagram of in vivo therapy in tumor-bearing mice. **b** Representative photographs of tumor-bearing mice after various treatments. **c** The photographs of all tumors taken from mice after various treatments. (Groups included (1) control, (2) PBS + NIR, (3) AIBI + NIR, (4) AHPR + NIR, (5) AHTPR + NIR). **d** Tumor volume growth curves of tumor-bearing mice during the administration of different formulations. **e** Tumor weights were measured after various treatments. **f** Body weight changes of MNNG/HOS tumor-bearing mice in different groups during the treatment. **g** H&E and immunohistochemical (TUNEL and Ki-67) analyses of tumor slices in different groups. (*P < 0.05, **P < 0.01, ***P < 0.001)

Inspired by the promising in vitro therapeutic effects and excellent tumor accumulation of the as-prepared NPs, a MNNG/HOS tumor xenograft model was established to further validate the in vivo LPTT/TDT synergistic therapeutic efficacy of the nanoplatforms. The experimental process in vivo was showed in Fig. 7a. As displayed in Fig. 7a–d, the tumors of mice without treatment and mice treated with PBS + NIR grew rapidly throughout the experiment, indicating that NIR irradiation alone had little inhibitory effect on the tumor growth. Comparatively, the mice treated with AIBI + NIR and AHTP + NIR showed slight tumor growth inhibition effect while the growth of tumors on mice treated with AHTPR + NIR was noticeably controlled, which might be contributed to the precise accumulation of AHTPR in tumor compared with nonspecific AIBI. Moreover, AHTPR could significantly improve the oxygen-irrelevant free radicals-based anticancer effect through reducing the intracellular GSH levels. Unsurprisingly, the mice injected with AHTPR couple with NIR irradiation exhibited more excellent tumor growth inhibitory efficacy, suggesting that selectively generation of free radicals in mitochondria might dramatically enhance the therapeutic efficacy. Notably, no significant body weight changes were observed (Fig. 7f) in all groups during the therapy, showing all treatments had no significant systemic toxicity to the animals.

At the end of the treatment, the tumor tissues and main organs of mice were collected for histopathological studies. As shown in Fig. 7g, the H&E staining of tumor sections demonstrated that AHTPR + NIR group exhibited the most significant necrosis. TUNEL staining also exhibited that AHTPR + NIR group caused cell apoptosis in a significantly higher level compared with groups. Moreover, the expression of antigen Ki‐67 was further investigated to evaluate the tumor cell proliferation. As expect, tumor cells in AHTPR + NIR group showed obviously lower expression of antigen Ki-67 compared to those in other groups. Additionally, the H&E staining images of major organs (heart, liver, spleen, lung, and kidney) demonstrated that no noticeable tissue damage was observed in those mice with various treatments (Additional file 1: Fig. S15). Meanwhile, the levels of the hepatic and renal function markers (ALT and BUN) were all found to be normal, indicating the favorable biosafety of these therapeutic strategies (Additional file 1: Fig. S16). These results collectively validated that the designed AHTPR nanoplatforms at a low temperature laser irradiation could eradicate tumors effectively without appreciable side effects to the treated animals.

## Conclusion

In summary, we successfully synthesized a biocompatible multifunctional nanoplatform composed of RGD functioned PDA as a shell and TPP modified H-mMnO_2_ as a core (AHTPR), which could achieve synergistic anticancer performance via the combination of mitochondria targeting LPTT/TDT. The promising delivery system was featured with distinctive advantages such as RGD induced active tumor-targeting, excellent mitochondria targeting ability, pH/NIR dual-responsive drug release, low temperature-activated alkyl radicals burst in mitochondria. The synergistic therapy efficacy was confirmed by effectively inducing cancer cell death in vitro and completely eradicating the tumors in vivo. Additionally, the excellent biosafety and biocompatibility of the nanoplatforms were confirmed both in vitro and in vivo. Taken together, the current study provides a novel paradigm toward oxygen-independent free-radical-based cancer therapy, especially for the treatment of hypoxic solid tumors.

## Supplementary Information

**Additional file 1: Figure S1.** TEM imaging of sSiO_2_ NPs. **Figure S2.** N_2_ adsorption/desorption isotherms and pore-size distribution curve of the as-synthesized H-mMnO_2_ NPs. **Figure S3.** EDS analysis of the AHTPR NPs. **Figure S4.** The dispersity and stability of the AHTPR NPs. a The dispersity of the AHTPR NPs was monitored in different media (water, α-MEM culture medium with or without 10% FBS) over a prolonged incubation time up to 7 days. b Hydrodynamic size variation of the AHTPR NPs dispersed in water, α-MEM culture medium with or without 10% FBS. **Figure S5.** Temperature variation curves of the different formulations (PBS, H-mMnO_2_, and HTPR) irradiated by an 808 nm laser (1 W·cm^−2^, 10 min). **Figure S6.** Photothermal-conversion performance of AHTPR NPs. (a) Photothermal performance of AHTPR NPs aqueous solution (120 μg mL^−1^) under 808 nm laser (1.0 W cm^−2^) irradiation. (b) Fitted linear relationship between time and − ln θ obtained from the cooling period of (a). **Figure S7.** The standard curve of AIBI determined by a UV–VIS spectrophotometer. **Figure S8.** The cellular uptake of ICG@HTPR NPs by MNNG/HOS cells under different conditions (chlorpromazine, genistein, amiloride, and cytochalasin D, 4 °C). Cells treated with ICG@HTPR NPs at 37 °C served as the control group. (* P < 0.05, ** P < 0.01, *** P < 0.001, N.S., not significant). **Figure S9.** Cell viability of MNNG/HOS cells after various treatments for 24 h. **Figure S10.** ESR spectra of DMPO in AIBI, HTPR (60 μg mL^−1^), and AHTPR (60 μg mL^−1^) solutions under 808 nm laser irradiation for 5 min. **Figure S11.** The corresponding surface plot images of free radicals in MNNG/HOS cells after various treatments for 24 h. **Figure S12.** TEM images of H-mMnO_2_ after exposure to different concentrations of GSH (0, 1, and 2 mM, respectively) at pH 6 for 30 min. **Figure S13.** XPS spectrum of the H-mMnO_2_ NPs in the presence of 2 mM GSH or not. a Mn2p spectrum of H-mMnO2 NPs. b Mn2p spectrum of H-mMnO_2_ NPs treated with GSH (2 mM) at pH 6.0. **Figure S14.** Relative protein levels of Bax and Bcl-2 in MNNG/HOS after various treatment. **Figure S15.** H&E-stained images of major organs (heart, liver, spleen, lung, and kidney) of MNNG-HOS bearing mice after the various treatments. **Figure S16.** Biosafety evaluation by blood biochemistry test. **a** Serum levels of ALT (liver function index). **b** Serum levels of BUN (kidney function index).

## Data Availability

The datasets supporting the conclusions of this article are included within the article and its additional file.
