# Supplementary material for: Mitochondria-targeted accumulation of oxygen-irrelevant free radicals for enhanced synergistic low-temperature photothermal and thermodynamic therapy
Source: J Nanobiotechnology. 2021 Nov 25;19:390. doi: 10.1186/s12951-021-01142-6 (PMC8620660; doi:10.1186/s12951-021-01142-6)
Supplement: Supplementary file 1 — Additional file 1: Figure S1. TEM imaging of sSiO2 NPs. Figure S2. N2 adsorption/desorption isotherms and pore-size distribution curve of the as-synthesized H-mMnO2 NPs. Figure S3. EDS analysis of the AHTPR NPs. Figure S4. The dispersity and stability of the AHTPR NPs. a The dispersity of the AHTPR NPs was monitored in different media (water, α-MEM culture medium with or without 10% FBS) over a prolonged incubation time up to 7 days. b Hydrodynamic size variation of the AHTPR NPs dispersed in water, α-MEM culture medium with or without 10% FBS. Figure S5. Temperature variation curves of the different formulations (PBS, H-mMnO2, and HTPR) irradiated by an 808 nm laser (1 W·cm−2, 10 min). Figure S6. Photothermal-conversion performance of AHTPR NPs. (a) Photothermal performance of AHTPR NPs aqueous solution (120 μg mL−1) under 808 nm laser (1.0 W cm−2) irradiation. (b) Fitted linear relationship between time and − ln θ obtained from the cooling period of (a). Figure S7. The standard curve of AIBI determined by a UV–VIS spectrophotometer. Figure S8. The cellular uptake of ICG@HTPR NPs by MNNG/HOS cells under different conditions (chlorpromazine, genistein, amiloride, and cytochalasin D, 4 °C). Cells treated with ICG@HTPR NPs at 37 °C served as the control group. (* P < 0.05, ** P < 0.01, *** P < 0.001, N.S., not significant). Figure S9. Cell viability of MNNG/HOS cells after various treatments for 24 h. Figure S10. ESR spectra of DMPO in AIBI, HTPR (60 μg mL−1), and AHTPR (60 μg mL−1) solutions under 808 nm laser irradiation for 5 min. Figure S11. The corresponding surface plot images of free radicals in MNNG/HOS cells after various treatments for 24 h. Figure S12. TEM images of H-mMnO2 after exposure to different concentrations of GSH (0, 1, and 2 mM, respectively) at pH 6 for 30 min. Figure S13. XPS spectrum of the H-mMnO2 NPs in the presence of 2 mM GSH or not. a Mn2p spectrum of H-mMnO2 NPs. b Mn2p spectrum of H-mMnO2 NPs treated with GSH (2 mM) at pH 6.0. Figure S1 [file 12951_2021_1142_MOESM1_ESM.docx]

**Additional Information**

**Mitochondria-targeted accumulation of oxygen-irrelevant free radicals for enhanced synergistic low-temperature photothermal and thermodynamic therapy**

Hongzhi Hu^1^^, 3, 4, *^, Xiangtian Deng^2, *^, Qingcheng Song^3, 4, *^, Wenbo Yang^1^, Yiran Zhang^2^, Weijian Liu^1, 3, 4^, Shangyu Wang^1^, Zihui Liang^5^, Xin Xing^3, 4^, Jian Zhu^2^, Junzhe Zhang^2. 3, 4^, Zengwu Shao^1, #^, Baichuan Wang^1, #^, Yingze Zhang^1, 3, 4, #^

^1^ Department of Orthopaedics, Union Hospital, Tongji Medical College, Huazhong University of Science and Technology, Wuhan 430022, China.

^2^ School of Medicine, Nankai University, Tianjin 300071, China.

^3^ Department of Orthopaedic Surgery, The Third Hospital of Hebei Medical University, Shijiazhuang 050051, China.

^4^ NHC Key Laboratory of Intelligent Orthopeadic Equipment, Third Hospital of Hebei Medical University, Shijiazhuang, Hebei, China.

^5^ Collaborative Innovation Center for Advanced Organic Chemical Materials Co-constructed by the Province and Ministry, Hubei University, Wuhan 430062, China.


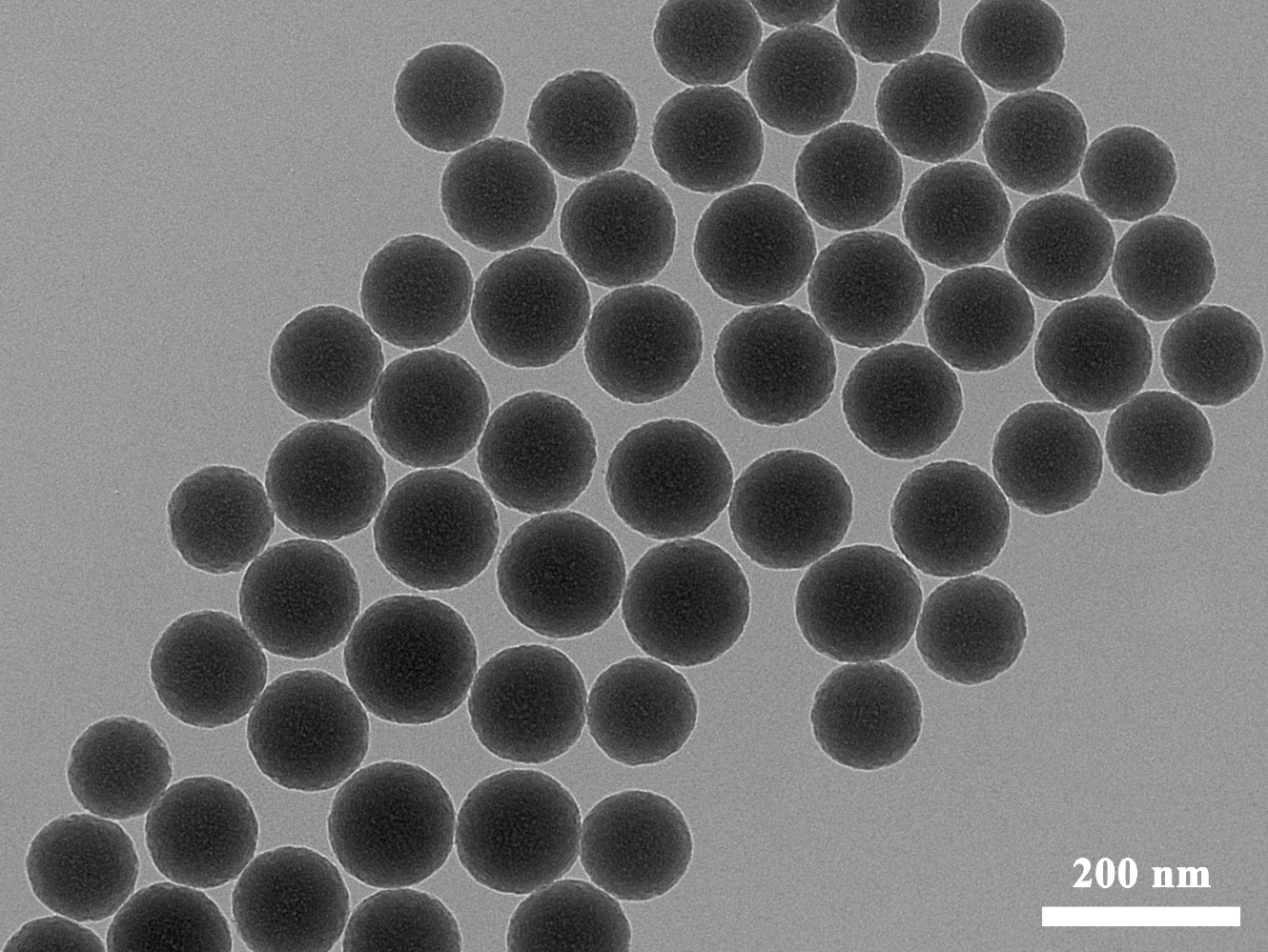


**Figure. S1** TEM imaging of sSiO_2_ NPs.


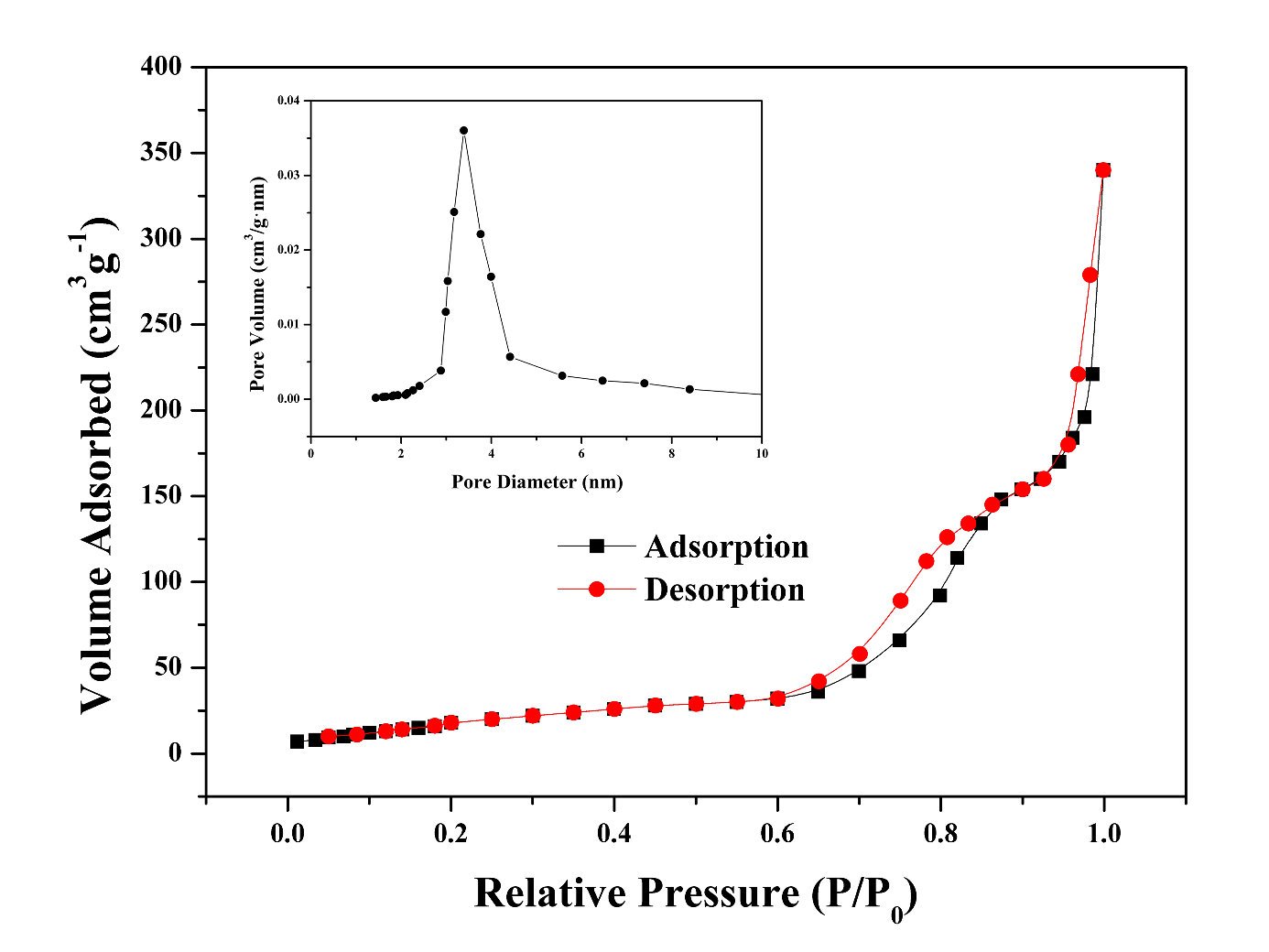


**Figure. S2** N_2_ adsorption/desorption isotherms and pore-size distribution curve of the as-synthesized H-mMnO_2_ NPs.


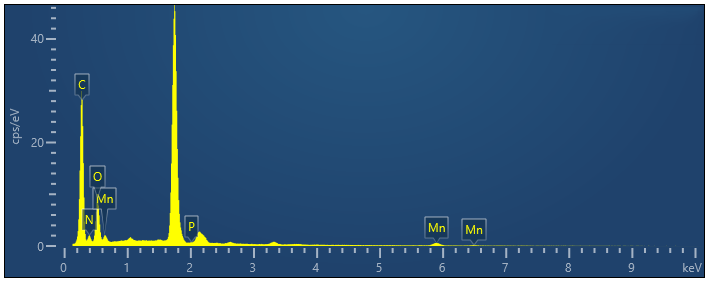


**Figure. S3** EDS analysis of the AHTPR NPs.


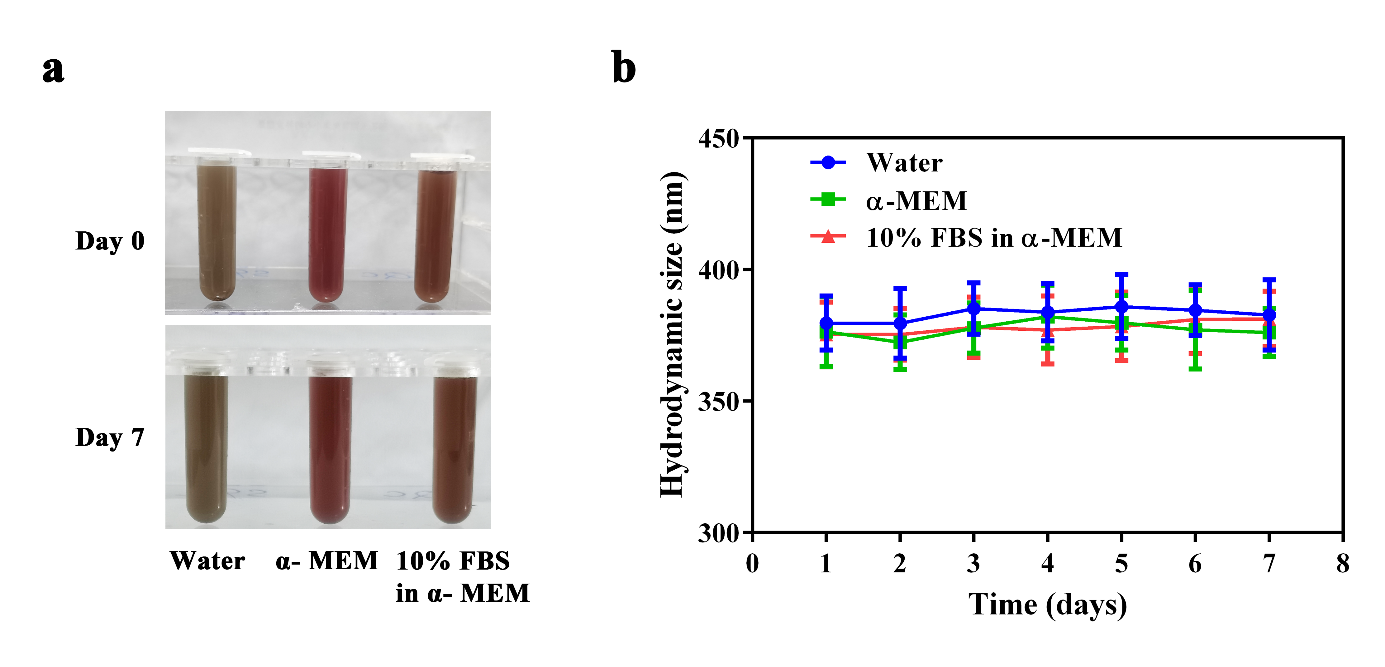


**Figure. S4** The dispersity and stability of the AHTPR NPs. a The dispersity of the AHTPR NPs was monitored in different media (water, α-MEM culture medium with or without 10% FBS) over a prolonged incubation time up to 7 days. b Hydrodynamic size variation of the AHTPR NPs dispersed in water, α-MEM culture medium with or without 10% FBS.

**
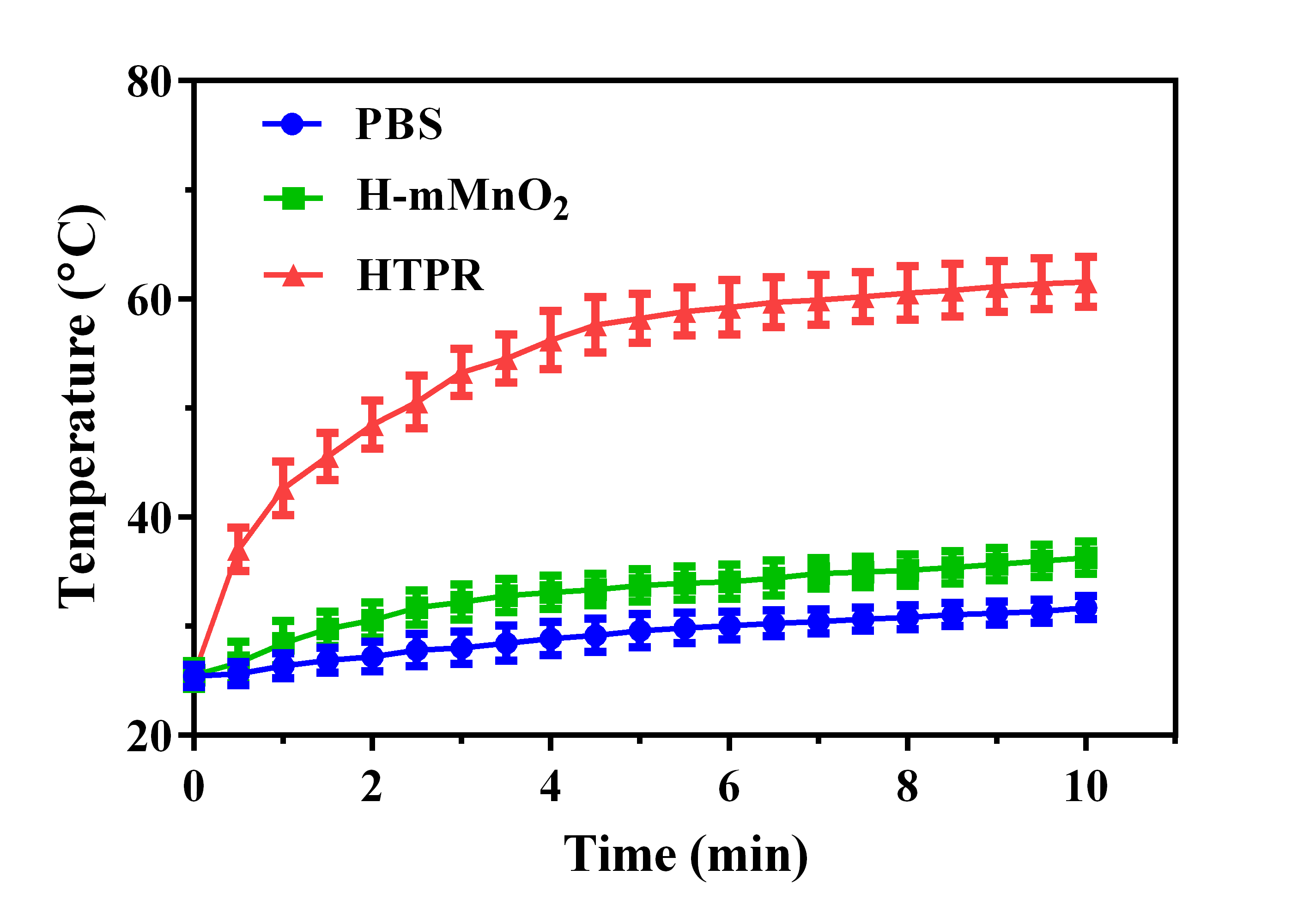
**

**Figure. S5** Temperature variation curves of the different formulations (PBS, H-mMnO_2_, and HTPR) irradiated by an 808 nm laser (1 W·cm^−2^, 10 min).


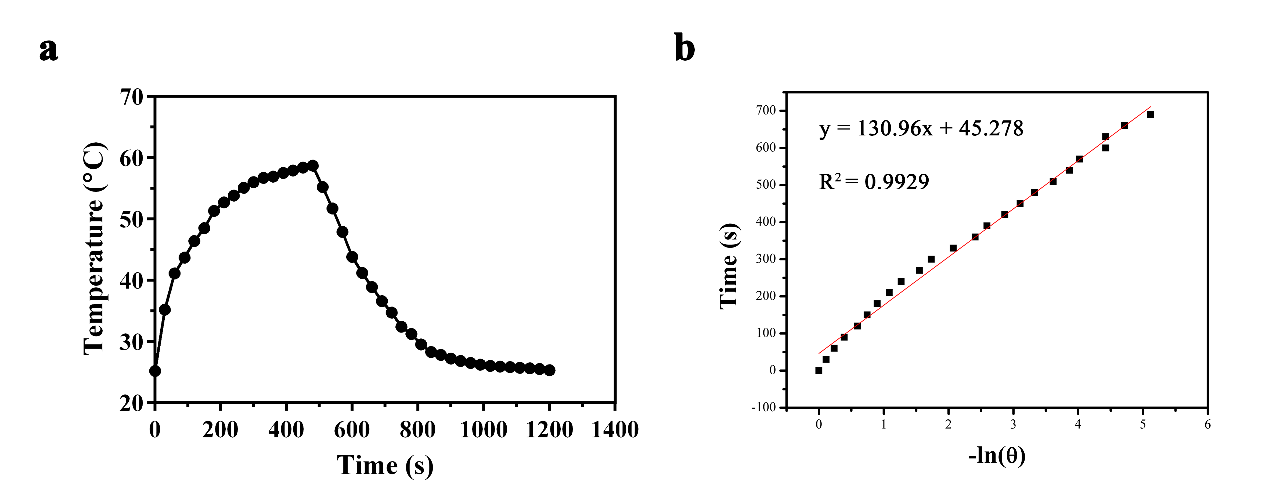


**Figure. S6** Photothermal-conversion performance of AHTPR NPs. (a) Photothermal performance of AHTPR NPs aqueous solution (120 μg mL^-1^) under 808 nm laser (1.0 W cm^-^^2^) irradiation. (b) Fitted linear relationship between time and −ln θ obtained from the cooling period of (a).


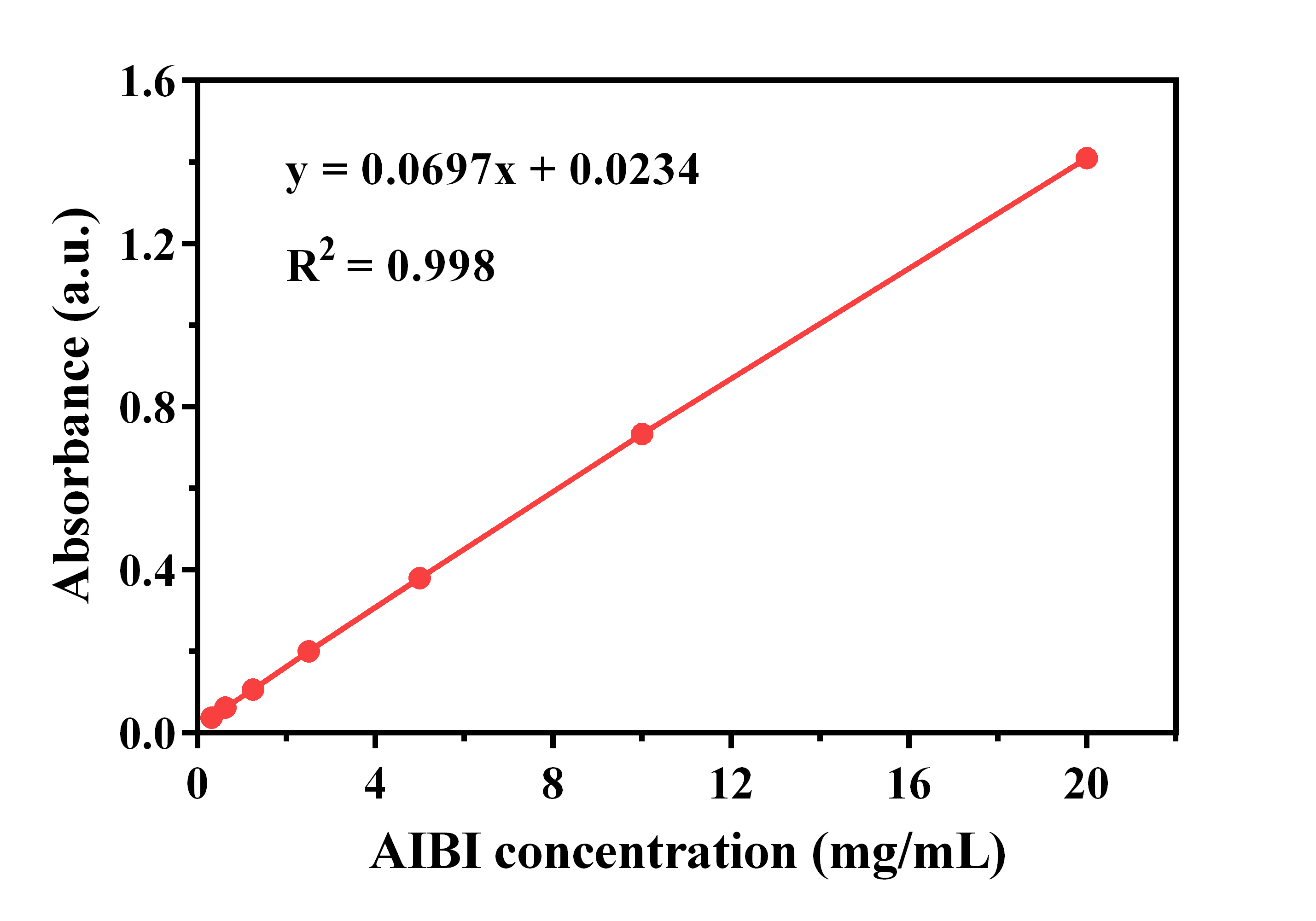


**Figure. S7** The standard curve of AIBI determined by a UV-VIS spectrophotometer.


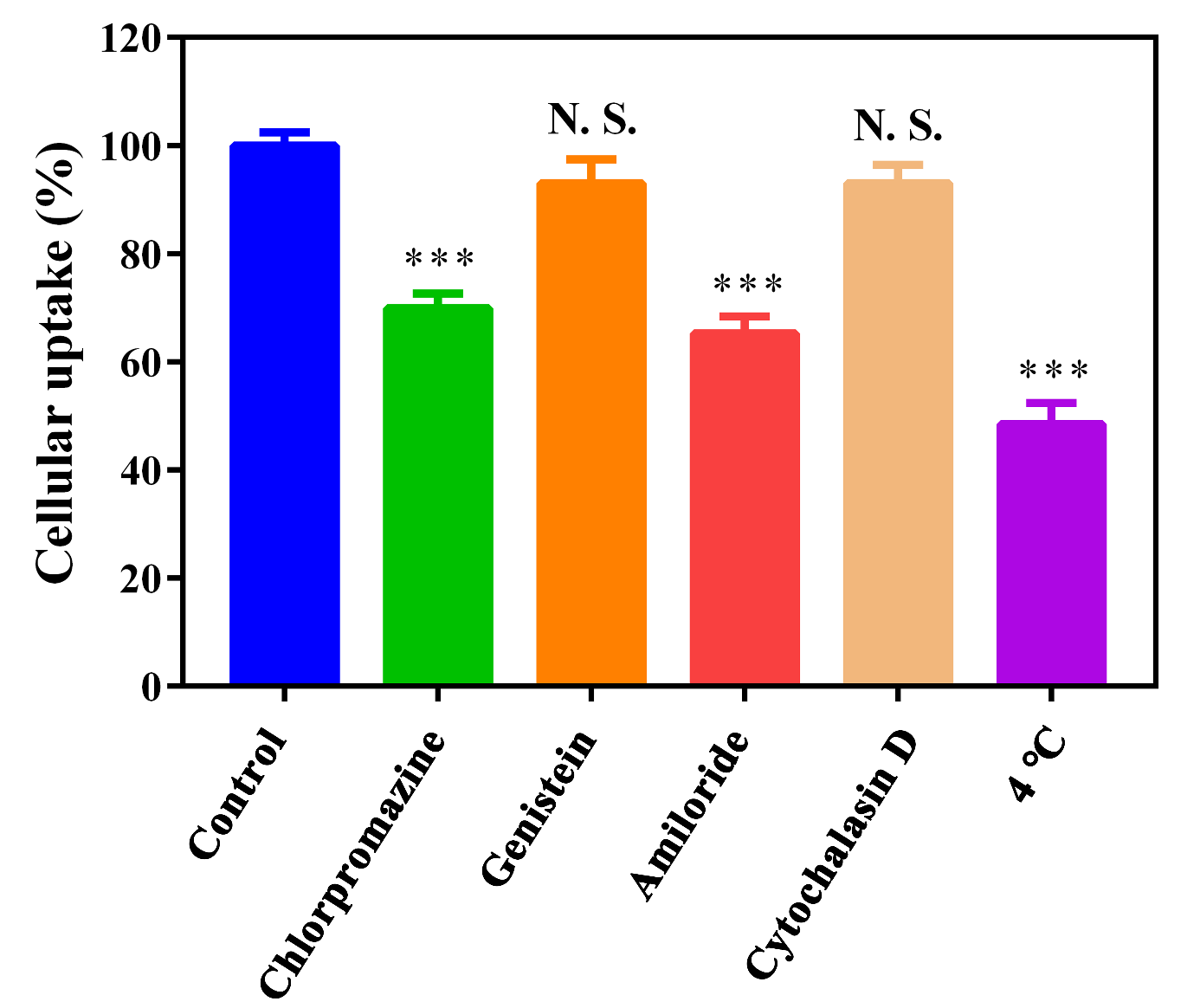


**Figure. S8** The cellular uptake of ICG@HTPR NPs by MNNG/HOS cells under different conditions (chlorpromazine, genistein, amiloride, and cytochalasin D, 4 °C). Cells treated with ICG@HTPR NPs at 37°C served as the control group. (* P < 0.05, ** P < 0.01, *** P < 0.001, N.S., not significant).


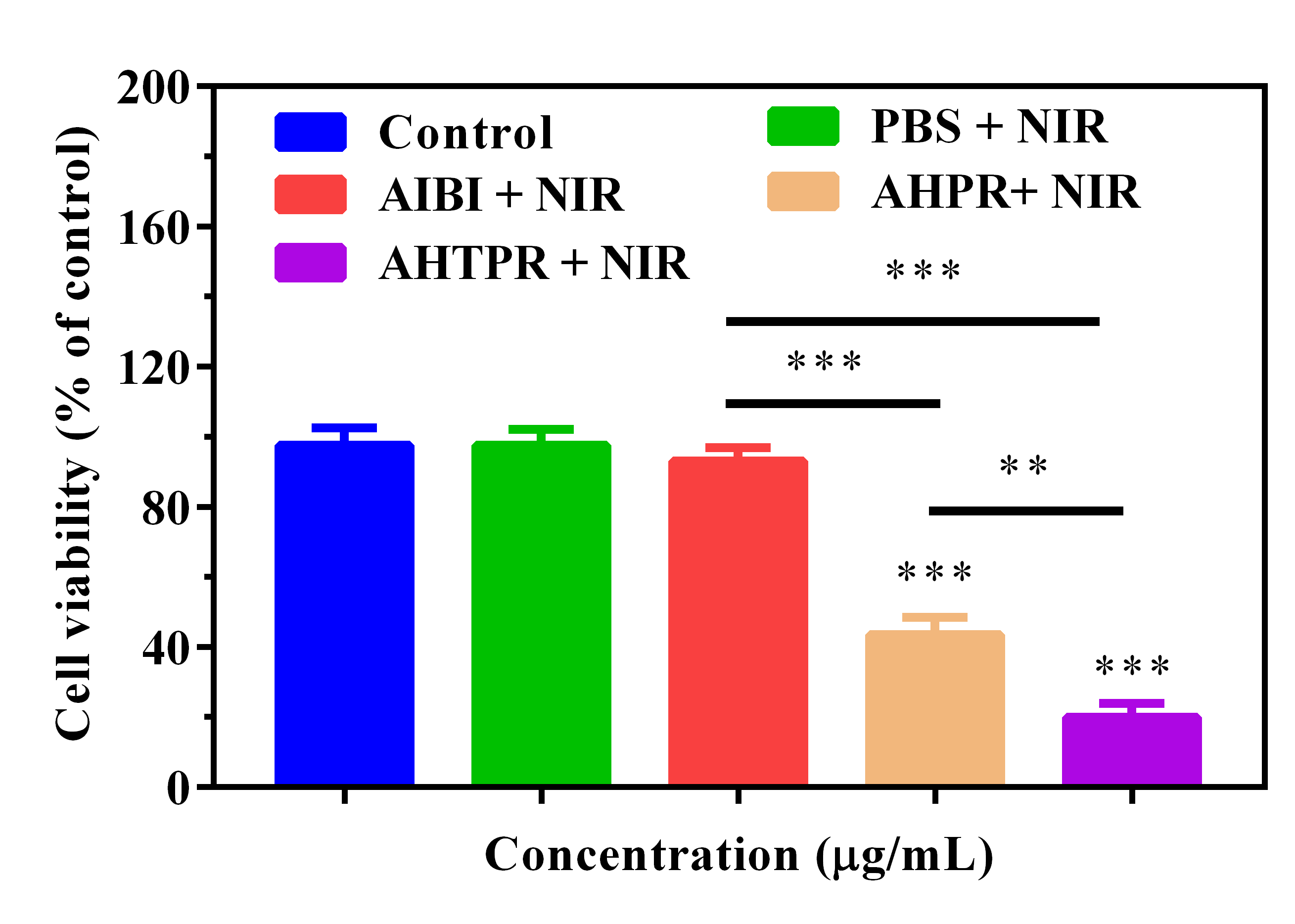


**Figure. S9** Cell viability of MNNG/HOS cells after various treatments for 24h.





**Figure. S10 ESR spectra of** **DMPO in** **AIBI, HTPR** **(60** μg mL^−1^**), and AHTPR (60** μg mL^−1^**) solutions under 808 nm laser irradiation for 5 min.**


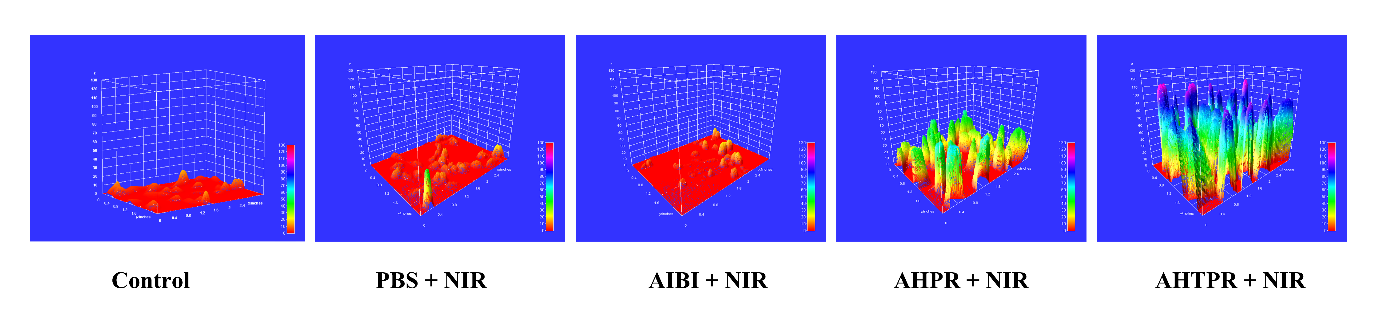


**Figure. S****11** The corresponding surface plot images of free radicals in MNNG/HOS cells after various treatments for 24h.


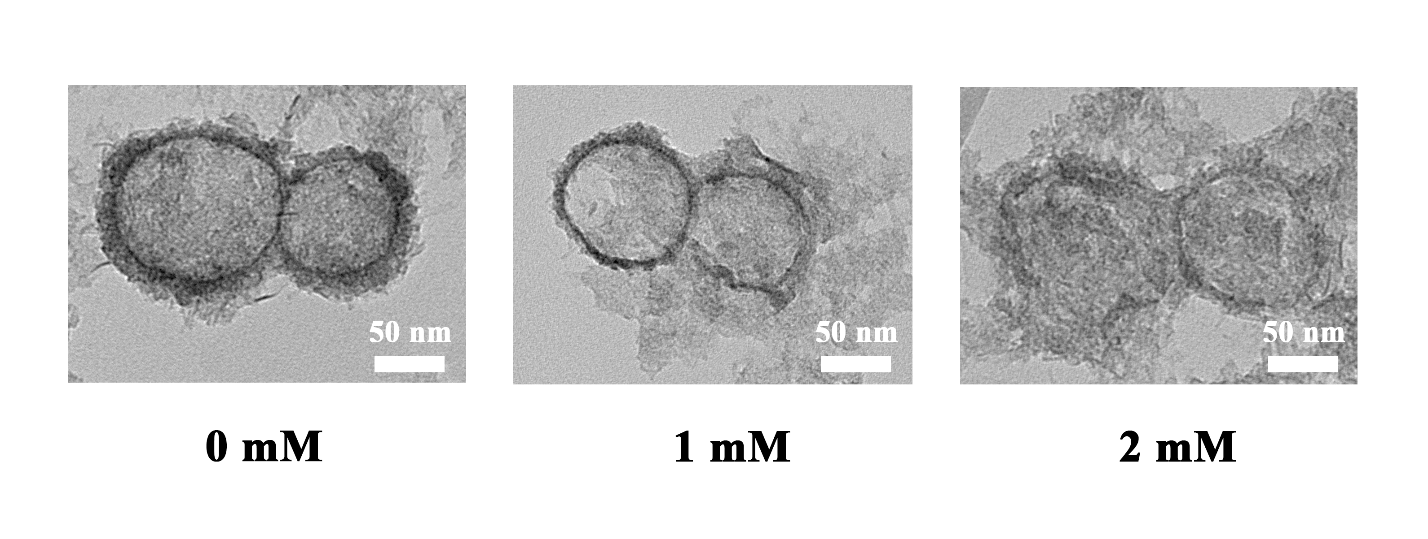


**Figure. S12** TEM images of H-mMnO_2_ after exposure to different concentrations of GSH (0, 1, and 2 mM, respectively) at pH 6 for 30 min.


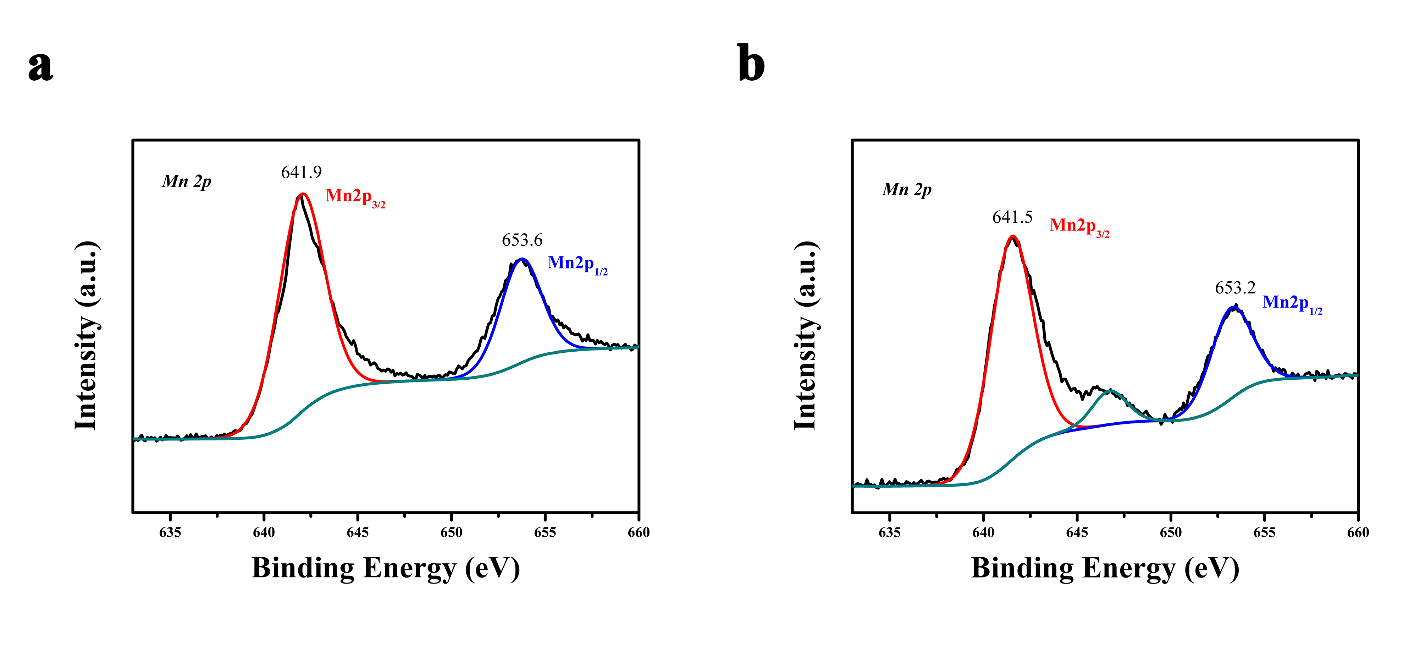


**Figure. S1****3** XPS spectrum of the H-mMnO2 NPs in the presence of 2 mM GSH or not. a Mn2p spectrum of H-mMnO2 NPs. b Mn2p spectrum of H-mMnO_2_ NPs treated with GSH (2 mM) at pH 6.0.


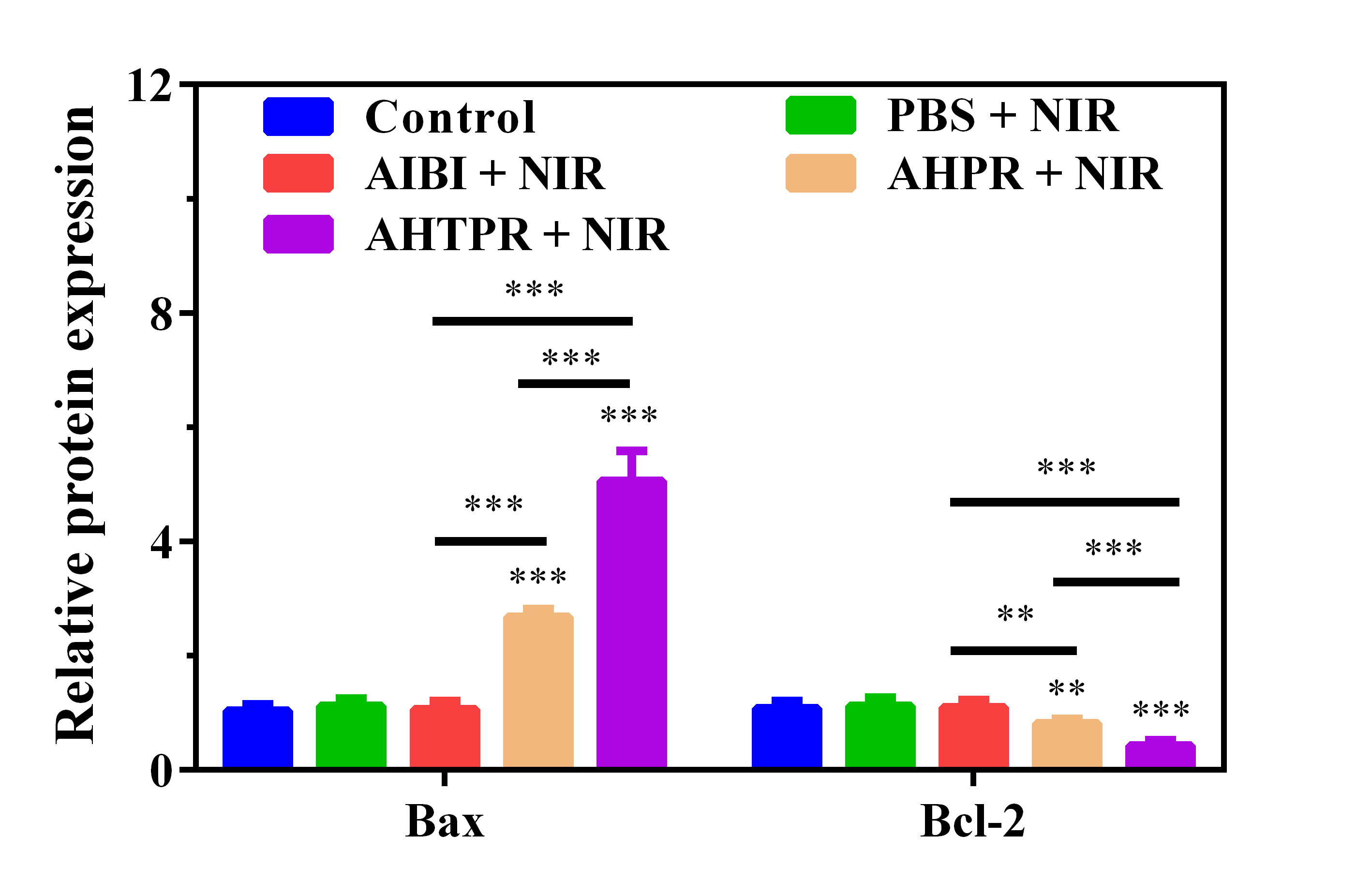


**Figure. S14** Relative protein levels of Bax and Bcl-2 in MNNG/HOS after various treatment.


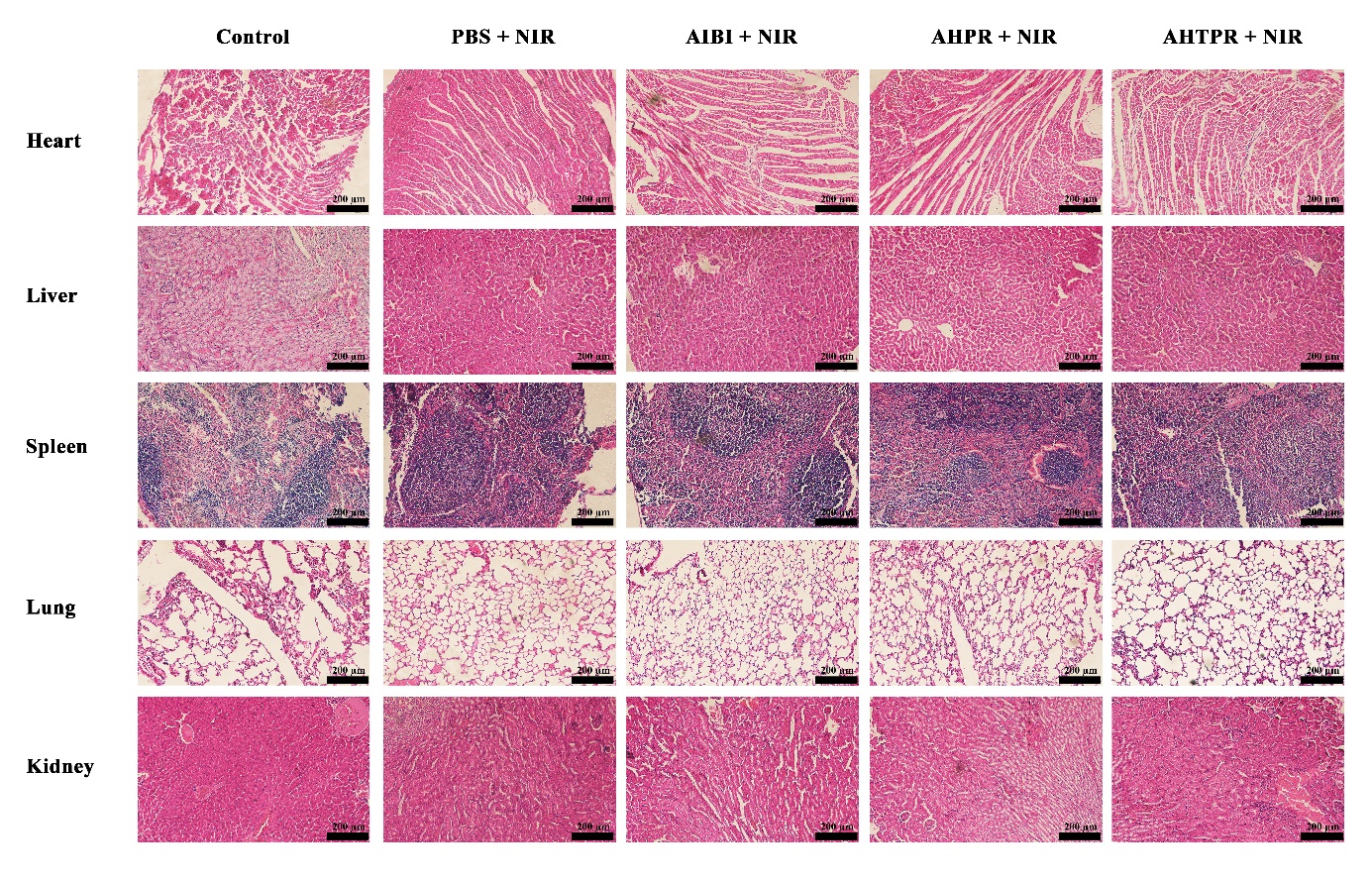


**Figure. S15** H&E-stained images of major organs (heart, liver, spleen, lung, kidney) of MNNG-HOS bearing mice after the various treatments.


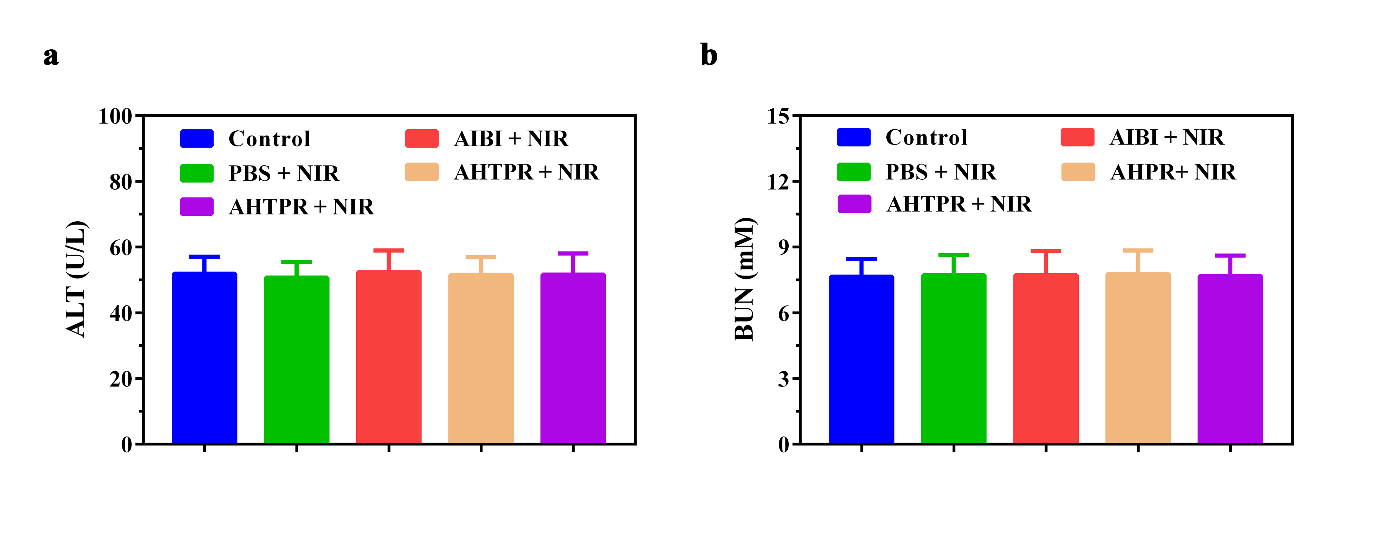


**Fi****gure. S16** Biosafety evaluation by blood biochemistry test. **a** Serum levels of ALT (liver function index). **b** Serum levels of BUN (kidney function index).
